# In Silico Investigation of the Anti-Tumor Mechanisms of Epigallocatechin-3-Gallate

**DOI:** 10.3390/molecules24071445

**Published:** 2019-04-11

**Authors:** Wang Wang, Xiuhong Xiong, Xue Li, Qinyang Zhang, Wentao Yang, Linfang Du

**Affiliations:** Key Laboratory of Bio-resources and Eco-environment of the Ministry of Education, College of Life Sciences, Sichuan University, Chengdu 610064, China; wangwang.jeff@foxmail.com (W.W.); xiongxiuhong0119@foxmail.com (X.X.); xueli.lai@foxmail.com (X.L.); zhangqy_wzmsly@foxmail.com (Q.Z.); yangwentao_sh@foxmail.com (W.Y.)

**Keywords:** EGCG, reverse docking, MD simulations, anti-tumor mechanism

## Abstract

The EGCG, an important component of polyphenol in green tea, is well known due to its numerous health benefits. We employed the reverse docking method for the identification of the putative targets of EGCG in the anti-tumor target protein database and these targets were further uploaded to public databases in order to understand the underlying pharmacological mechanisms and search for novel EGCG-associated targets. Similarly, the pharmacological linkage between tumor-related proteins and EGCG was manually constructed in order to provide greater insight into the molecular mechanisms through a systematic integration with applicable bioinformatics. The results indicated that the anti-tumor mechanisms of EGCG may involve 12 signaling transduction pathways and 33 vital target proteins. Moreover, we also discovered four novel putative target proteins of EGCG, including IKBKB, KRAS, WEE1 and NTRK1, which are significantly related to tumorigenesis. In conclusion, this work may provide a useful perspective that will improve our understanding of the pharmacological mechanism of EGCG and identify novel potential therapeutic targets.

## 1. Introduction

Green tea that is produced from *Camellia sinensis* plant leaves is one of the most extensively consumed beverages all over the world and has been widely used in various clinical trials due to its antitumor [1,2], anticancer [2,3,4], antiinflammation [5,6,7,8] and antiangiogenesis effects [9,10,11]. In recent years, it has shown quite promising results in the prevention of cancer [4,6]. The chemo-preventive effects of green tea are mediated by its polyphenols, such as Epigallocatechin-3-gallate (EGCG), EGC (Epigallocatechin), ECG (Epicatechin-3-gallate), EC (Epicatechin), GCG (Gallocatechin-3-gallate), CG (Catechin-3-gallate), GC (Gallocatechin) and catechin [12]. The EGCG, an important catechin in green tea, has also a therapeutic role and can be used against a number of health-damaging diseases, particularly diabetes, Parkinson, Alzheimer, stroke and obesity [4,8,13].

EGCG prevents cancer by suppressing tumor-associated proteins and modulating other signaling pathways involved in tumor development [4,6,13,14]. Furthermore, previous research demonstrated that EGCG effectively induces apoptosis and cell cycle arrest during tumorigenesis through regulation of NF-*k*B [15], cyclin D1 [4], p21/WAF1/CIP1 [16], p27/KIP1 [16] and cyclin-dependent kinases (CDK) [4,17]. Moreover, in vitro investigations have indicated that EGCG suppresses AKT and ERK phosphorylation, whereas it also enhances the activation of FOXO transcription factors, which results in cell cycle arrest and apoptosis [18]. In addition, EGCG binds to the 67-kDa laminin receptor (67LR) and hinders its expression during carcinogenesis [19,20]. Similarly, it also inhibits MAPK (mitogen-activated protein kinase) activity and is involved in the regulation of various important signaling pathways, including JNK and RAS signaling [4,16]. On the other hand, it suppresses the activity of Pin1 in order to modulate multiple signaling pathways, such as Wnt-β-catenin and NF-kB signaling [21,22,23]. A number of preclinical studies suggested that EGCG treatment decreases the incidence and diversity of tumors in various organs, such as liver, stomach, skin, lung, breast and colon [4,24]. Therefore, the discovery of the novel EGCG-related targets and the illustration of their molecular mechanisms of action would be very meaningful [4,6,13].

Despite gaining such remarkable scientific attention over the last years, little is known about the potential targets of EGCG and their molecular mechanisms [12]. With the advent of computational chemistry, structural biology and bioinformatics, the reverse docking method, a powerful tool for drug repositioning and rescue, has been frequently applied to facilitate the discovery of new targets or receptors [25,26]. Traditional docking involves one target–many ligands, while the reverse docking is one ligand–many targets, in which the ligand is docked against an array of relevant targets and is ranked according to ‘some score’ [27]. Therefore, several reverse docking tools, such as TarFisDock, idTarget, INVDOCK or conventional docking softwares (Autodock, Autodock Vina, DOCK, Glide and Ledock), have been successfully developed and explored in the last few years [28]. Some researchers have applied INVDOCK to predict the potential targets of the broad-spectrum anticancer drug BBR [29,30]. Zhang et al. employed TarFisDock for the rapid identification of bioactive compounds and their targets from medicinal plants [31]. Investigators have identified the potential antineoplastic targets of tea polyphenols using Autodock 4 and TarFisDock based on a comparative reverse docking strategy [12]. Wang et al. adopted a reverse docking method to predict human nuclear receptors of environmental contaminants by means of Autodock Vina software [32]. Chen et al. engaged Ledock and Autodock Vina for the prediction of the potential anti-tumor activity of marine compounds [26]. Park et al. recognized potential targets for ginsenosides by applying the Glide software [33]. Li et al. implemented a reverse docking approach for the demonstration of the pharmacological mechanism of *G. biloba* in the case of Alzheimer’s disease [25]. Additionally, various scientific databases, such as KEGG [34], DAVID [35] and STRING database [36], have been generally used to explore the targets related to the possible molecular mechanisms.

**Figure 1:**
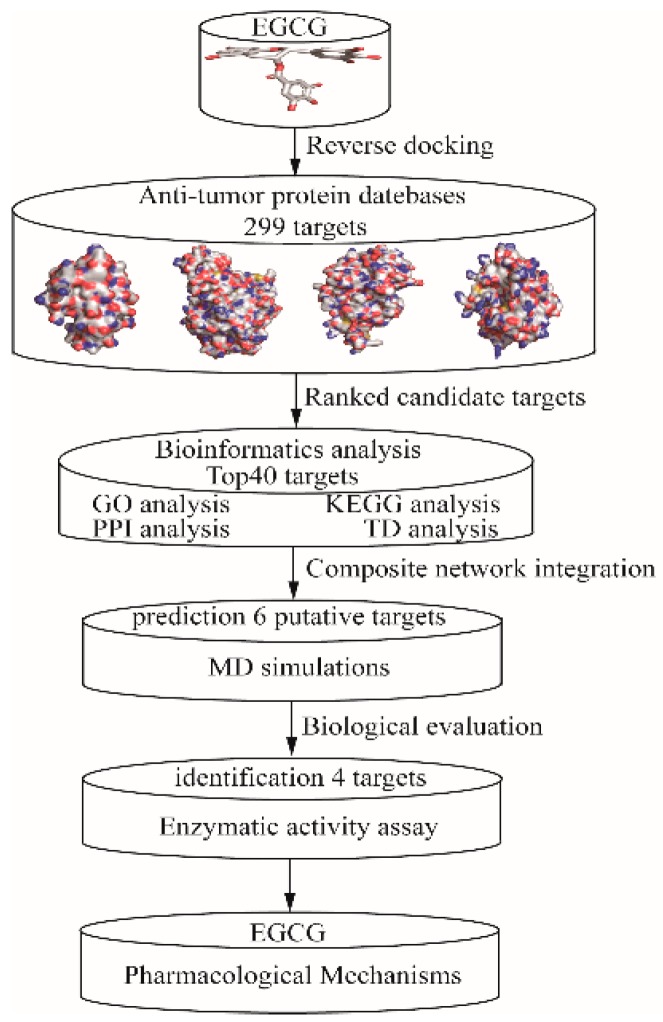
The schematic representation of the epigallocatechin gallate (EGCG) anti-tumor mechanisms. GO, KEGG, PPI, TP and MD presented gene ontology, Kyoto encyclopedia of genes and genomes, protein–protein interaction, target–pathway and molecular dynamics.

In the present study, we integrated bioinformatics and computational chemistry techniques, including the utilization of public databases, reverse docking and MD simulations, in order to describe a novel paradigm for constructing easily interpretable networks. In short, our finding provides new insight into our understanding of the pharmacological mechanism of EGCG and its role in the prevention of carcinogenesis. Figure 1 demonstrates the schematic and overall result from each step in this scientific search.

## 2. Results

### 2.1. Target Proteins Database and Reverse Virtual Screening

**Figure 2:**
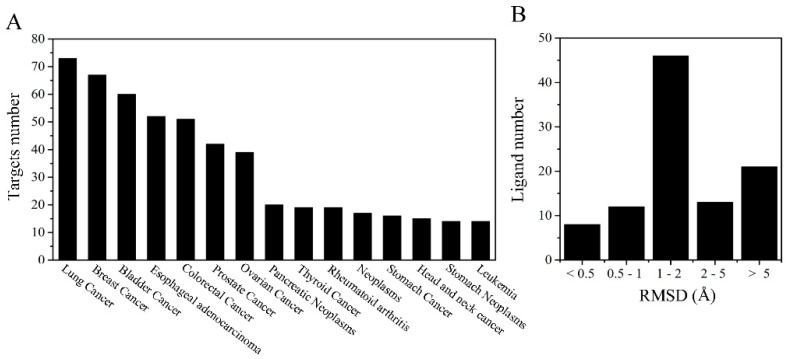
The corresponding disease of the target proteins set (**A**) and the distribution of the RMSD values for redocked ligands using AutoDock Vina (**B**).

All the 299 proteins were installed in the anti-tumor target proteins database and annotated into the GAD (Genetic Association Database) in the DAVID 6.8 package [35]. The corresponding disease of the set of target proteins contained 360 diseases entries (*p*-value ≤ 0.05) and the top 15 entries are shown in Figure 2A and listed in Table S1, respectively. Diseases related to the tumor and cancer in our target database are lung cancer, breast cancer, bladder cancer, esophageal adenocarcinoma, colorectal cancer, prostate cancer, ovarian cancer, pancreatic neoplasms, thyroid cancer, neoplasms, stomach cancer, head and neck cancer, stomach neoplasms and leukemia. Simultaneously, 271 target proteins are annotated into GAD at an enrichment rate of 91.25%, which suggests that most target proteins are enriched in the disease databases.

Before applying the formal reverse virtual screening, redocking was carried out to validate the accuracy of the docking program [26]. As seen in Figure 2B, the number of RMSD values was 8 (less than 0.5 Å), 12 (between 0.5 and 1 Å), 46 (between 1 and 2 Å), 13 (between 2 and 5) and 21 (greater than 5 Å), respectively. As the RMSD value for the heavy atoms of the ligand is less than 2 Å, this suggests that the parameters and the scoring algorithms are reasonable [26,37]. Moreover, according to the Autodock Vina, the RMSD value of 66 ligands was less while that of 34 ligands was greater than 2 Å. The similarity of the results to that of previous studies suggests that the parameters and the scoring algorithms are relatively reliable while using the Autodock Vina software [38].

**Figure 3:**
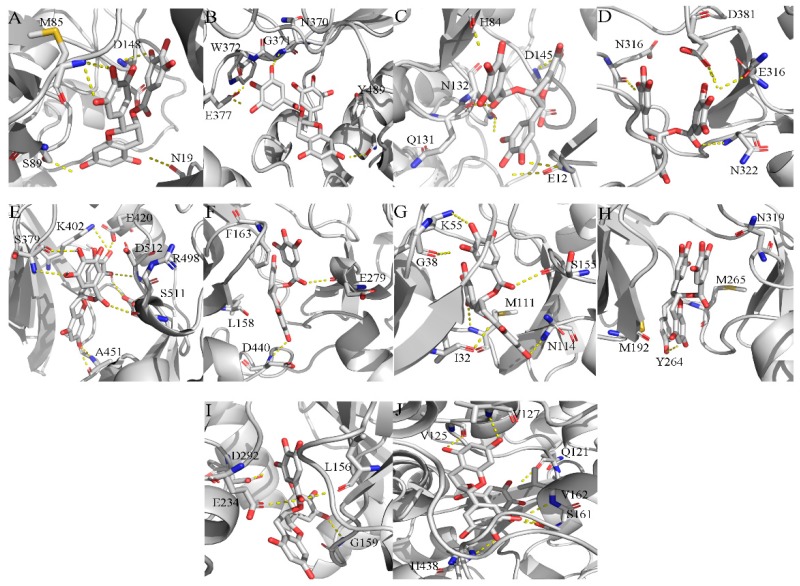
Analysis of binding modes between EGCG and known targets based on the reverse docking results from ranking targets. (**A**) Binding mode of proto-oncogene tyrosine-protein kinase FYN (PDB ID: 2DQ7). (**B**) Binding mode of nitric oxide synthase 2 (NOS2, PDB ID: 4NOS). (**C**) Binding mode of cyclin-dependent kinase 2 (CDK2, PDB ID: 2IW9). (**D**) Binding mode of tyrosine-protein kinase ABL1 (ABL1, PDB ID: 2V7A). (**E**) Binding mode of spleen tyrosine kinase (SYK, PDB ID: 1XBC). (**F**) Binding mode of serine-threonine protein kinase AKT2 (PDB ID: 2JDR). (**G**) Binding mode of mitogen-activated protein kinase 8 (MAPK8 or JNK1, PDB ID: 3ELJ). (**H**) Binding mode of interleukin-1 receptor-associated kinase 4 (IRAK4, PDB ID: 2NRU). (**I**) Binding mode of serine-threonine protein kinase AKT1 (PDB ID: 3MVH). (**J**) Binding mode of apoptotic protease activating factor 1 (APAF1, PDB ID: 1Z6T).

For the identification of EGCG anti-tumor efficiency, we performed reverse virtual screening with a database of 299 target proteins. The detailed results are listed in Table S2. The top 40 target proteins were selected based on their docking score (Table 1). In addition, 10 target proteins were identified as an inhibitor in previous investigations [2,4,14,24]. The binding modes of the 10 reported targets with EGCG, including FYN-EGCG [39], NOS2-EGCG [40,41], CDK2-EGCG [42,43], ABL1-EGCG [44], SYK-EGCG [8], AKT2-EGCG [1,45], MAPK8-EGCG [46], IRAK4-EGCG [47], AKT1-EGCG [1,45] and APAF1-EGCG [48], are shown in Figure 3. As observed, all target EGCG compounds were posed into the binding site and form extensive interactions with the key residues.

According to Figure 3A, EGCG interacts with the key residues N19, M86, S89 and D148 and forms four hydrogen bonds in the binding sites of proto-oncogene tyrosine-protein kinase FYN (PDB ID: 2DQ7). Similarly, the intermolecular interactions between EGCG and nitric oxide synthase 2 (NOS2, PDB ID: 4NOS) is involved in hydrophobic interactions with the key residues N370, G371, W372, E377 and Y489 and forms four hydrogen bonds (Figure 3B). Meanwhile, EGCG enters the hydrophobic pocket of cyclin-dependent kinase 2 (CDK2, PDB ID: 2IW9) and forms six hydrogen bonds with the key residues E12, H84, Q131, N132 and D145 (Figure 3C). The key residues E316, N322, N368 and D381 interact in a hydrophobic manner with EGCG and develop four stable hydrogen bonds in tyrosine-protein kinase ABL1 (ABL1, PDB ID: 2V7A, Figure 3D). At the same time, EGCG relocates to the active sites of spleen tyrosine kinase (SYK, PDB ID: 1XBC) and it establishes nine hydrogen bonds with the important residues S379, K402, E420, A451, R498, S511 and D512 (Figure 3E). The hydroxyl groups of EGCG forms two hydrogen bonds with the critical residues E279 and D4400 and it is involved in the hydrophobic interactions with the residues L158 and F163 in serine-threonine protein kinase AKT2 (PDB ID: 2JDR, Figure 3F). Moreover, the hydroxyl groups of EGCG also can form six hydrogen bonds by interacting with the residues I32, G38, K55, N114 and S155 in mitogen-activated protein kinase 8 (MAPK8 or JNK1, PDB ID: 3ELJ, Figure 3G). EGCG forms only one hydrogen bond with the residue Y264 and hydrophobic interactions with the residues M192, M265 and N319 in interleukin-1 receptor-associated kinase 4 (IRAK4, PDB ID: 2NRU, Figure 3H). EGCG inserts into the binding site of serine-threonine protein kinase AKT1 (PDB ID: 3MVH) with a hydroxyl group and forms four hydrogen bonds with the important residues L156, G159, E234 and D292 (Figure 3I). Similarly, the interactions between EGCG and apoptotic protease activating factor 1 (APAF1, PDB ID: 1Z6T) involves the formation of extensive hydrogen bonds with the residues Q121, V125, V127, S161, V162 and H438 (Figure 3J).

### 2.2. GO Analysis and KEGG Pathway Enrichment

**Figure 4:**
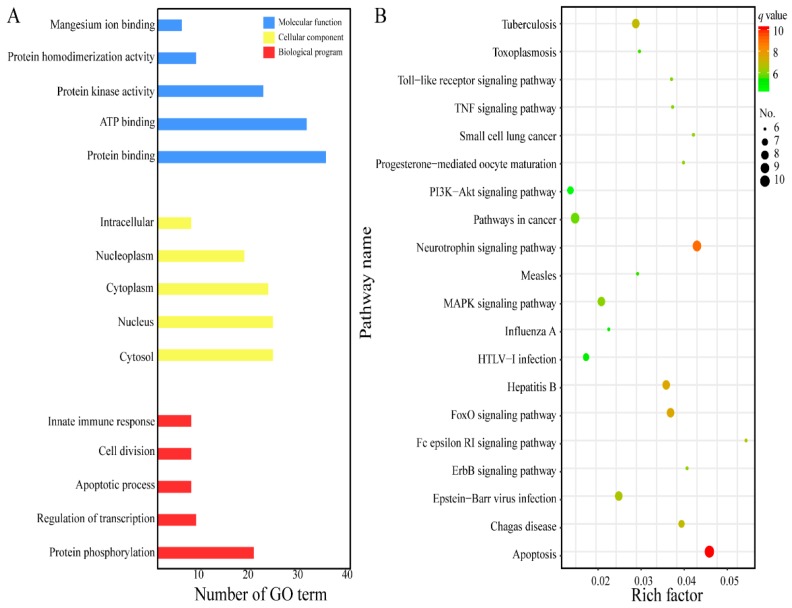
GO and KEGG enrichment analysis of top 40 target proteins. (**A**) The top 5 GO enrichment. GO, gene ontology. (**B**) Top 20 according to the KEGG pathway (KEGG, Kyoto encyclopedia of genes and genomes). The size and color of points represents the number of target proteins and the q-values, respectively. The rich factor showed the enrichment degree in KEGG pathway.

In the present research, the top 40 putative target proteins were annotated according to the GO term and KEGG pathway [25,26]. The GO term was divided into three functional parts: molecular function, biological process and cellular component [49]. As shown in Figure 4A, the molecular function mainly contains protein binding, ATP binding, protein kinase activity, protein homodimerization activity and magnesium ion binding; the cellular component is related to cytosol, nucleus, cytoplasm, nucleoplasm and intracellular space; and the biological program is involved in protein phosphorylation, regulation of transcription, apoptosis process, cell division and innate immune response.

Based on the *p*-value ≤ 0.05, the top 40 putative target proteins were mapped to the reference pathway in the KEGG database [34] and were involved in 98 pathways (Table S3). The bubble diagram was employed in Figure 4B to reveal the result of the complicated KEGG pathway enrichment. We observed that these signaling pathways are evidently related with human tumors or cancer, including small cell lung cancer as they are involved in related processes, such as apoptosis, neurotrophin, FoxO, MAPK, PI3K/Akt, ErbB, TNF and Toll-like receptor signaling pathway. Previous literature demonstrated that EGCG can effectively suppress carcinogenesis by modulating various signaling pathways, such as apoptosis, FoXO, ErbB, JAK/STAT, MAPK, PI3K/AKT, Wnt, AMPK and Notch [2,4,6,13,24]. Combining this information with the above KEGG analysis, we can conclude that the anti-tumor mechanism of EGCG is extremely complicated and involves multiple signaling pathways.

### 2.3. Analysis of Pharmacological Network

**Figure 5:**
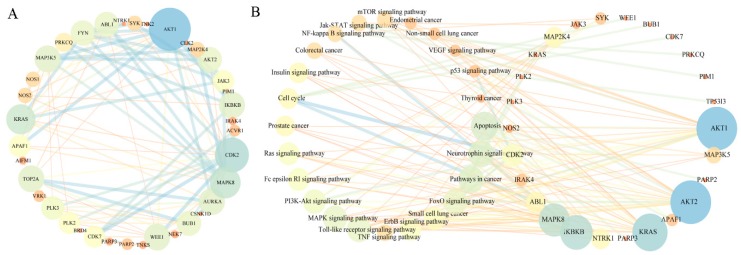
Analysis of pharmacological network. (**A**) PPI (protein–protein interaction) network created by uploading the top 40 target proteins to STRING database. The larger circle that represents the target proteins is considered to contain the vital ones. (**B**) TP (Target–pathway) network based on the KEGG pathway. The size of circle represents the importance degree for the target protein and pathway.

To further explore the relationship of complex pathways, the PPI network was prepared by uploading the top 40 putative target proteins to the STRING database [25,36]. A total of 37 putative target proteins were mapped to the STRING database and the results were displayed by using Cytoscape tools (Figure 5A). By observing the PPI (protein–protein interaction) network, we can conclude that AKT1, CDK2, MAPK8, KRAS, TOP2A, MAP3K5 play a significant role in relative signaling pathways, such as MAPK, AKT/PI3K, Ras and cell cycle. Additionally, IKBKB, AURKA, BUB1, WEE1, APAF1, ABL1, FYN and PRKCQ are also important and this is consistent with their molecular function.

Based on the results of KEGG pathway, the TP (Target–pathway) network containing 50 nodes and 132 edges was constructed (Figure 5B). The important signaling pathway includes apoptosis, neurotrophin, FoxO, ErbB, TNF, MAPK and PI3K/AKT. Similarly, these pathways are significantly associated with relative target proteins: AKT1, AKT2, KRAS, IKBKB, MAPK8, MAP3K5, APAF1, NTRK1 [4,24].

### 2.4. Exploration and Identification of the Potential Target Proteins

After connecting PPI with TP network, we observed that the target proteins, such as AKT1/2, KRAS, MAPK8, CDK2, IRAK4, IKBKB, MAP3K5, APAF1, JAK3, WEE1 and NTRK1, play a relatively important role in relative anti-tumor pathways. However, six relative targets, including IKBKB, MAP3K5, KRAS, JAK3, WEE1 and NTRK1, were not verified by the scientific methods. MD simulations are a powerful and effective method to validate relative target–EGCG compounds [50]. Therefore, based on the conformation of reverse docking, the MD simulations of six target–EGCG compounds were carried out in this research.

**Figure 6:**
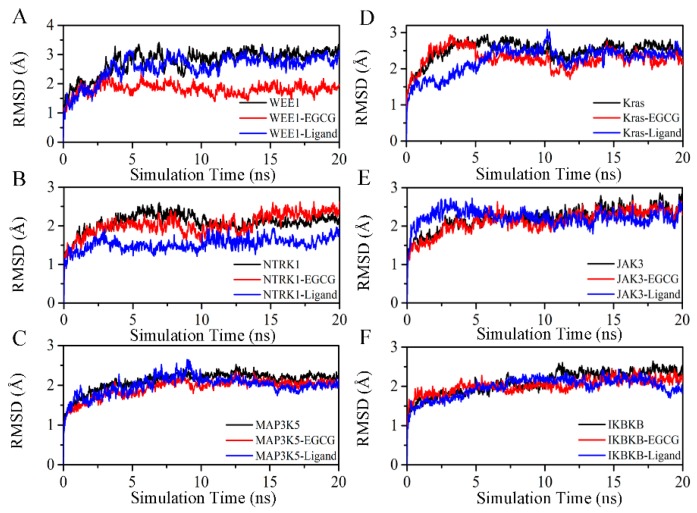
The backbone Cα-RMSD values during the 20 ns MD simulation. The black, red and blue lines show the backbone Cα-RMSD values of target proteins, target–EGCG complexes and target–ligand complexes, respectively. (**A**) The backbone Cα-RMSD values of WEE1. (**B**) The backbone Cα-RMSD values of NTRK1. (**C**) The backbone Cα-RMSD values of MAP3K5. (**D**) The backbone Cα-RMSD values of KRas. (**E**) The backbone Cα-RMSD values of JAK3. (**F**) The backbone Cα-RMSD values of IKBKB.

The assessment of the backbone Cα-RMSD value for each target–EGCG system is used to measure the structural stability in the MD simulation [51,52] as shown in Figure 6. As shown, most of the systems reached equilibrium after 5 ns when running a MD simulation with an average backbone Cα-RMSD of about 3 Å. Furthermore, we also observed that the backbone Cα-RMSD values of target–EGCG systems are less or equal to the targets and target–original ligand complexes during last 10 ns MD simulation, suggesting that EGCG are tightly bound to these targets and block the enzymatic activities.

**Figure 7:**
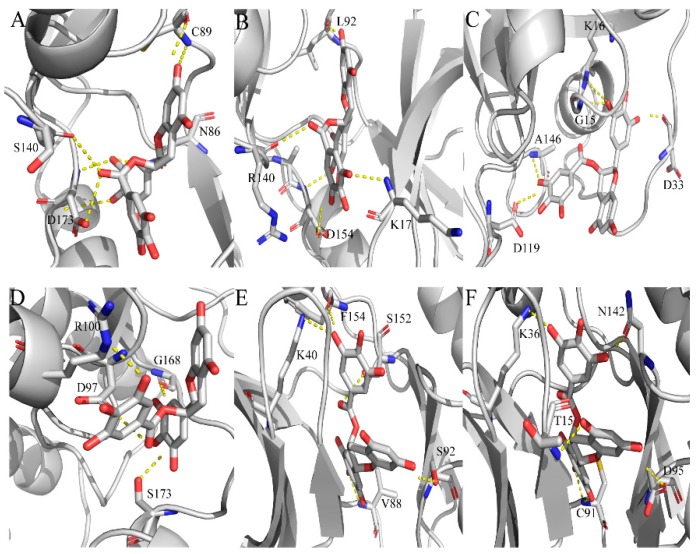
Analysis of binding modes between EGCG and potential target proteins. (**A**) Binding mode of mitosis inhibitor protein kinase WEE1 (PDB ID: 1X8B). (**B**) Binding mode of tyrosine-protein kinase JAK3 (PDB ID: 1YVJ). (**C**) Binding mode of kirsten rat sarcoma viral oncogene (KRAS, PDB ID: 3GFT). (**D**) Binding mode of high affinity nerve growth factor receptor (NTRK1, PDB ID: 4AOJ). (**E**) Binding mode of mitogen-activated protein kinase kinase kinase 5 (MAP3K5, PDB ID: 2CLQ). (**F**) Binding mode of inhibitor of nuclear factor kappa-B kinase subunit beta (IKBKB, PDB ID: 4KIK).

Based on the above MD results, the potential binding models of these target–EGCG compounds were further analyzed (Figure 7). Figure 7A shows that EGCG is stabilized in the binding site of mitosis inhibitor protein kinase WEE1 (PDB ID: 1X8B) through six hydrogen bonds with C89, N86, S140 and S140. On the other hand, EGCG interacted with tyrosine-protein kinase JAK3 (PDB ID: 1YVJ) and constructs five hydrogen bonds with the key residues K17, L92, R140 and D154 (Figure 7B). Similar to the binding mode of KRAS-EGCG compounds (KRAS, kirsten rat sarcoma viral oncogene; PDB ID: 3GFT), EGCG is stabilized in the binding site through five hydrogen bonds with the key residues G15, K16, D33, D1189 and A146 (Figure 7C). Meanwhile, EGCG enters the hydrophobic pocket of high affinity nerve growth factor receptor (NTRK1, PDB ID: 4AOJ) and forms four hydrogen bonds with the critical residues D97, R100, G168 and S173 (Figure 7D). EGCG enters the hydrophobic pocket of mitogen-activated protein kinase kinase kinase 5 (MAP3K5, PDB ID: 2CLQ) and forms five hydrogen bonds with the key residues K40, V88, S92, S152 and F154 (Figure 7E). Likewise, as shown in Figure 7F, EGCG interacts with the key residues T15, K36, C91, D95 and N142 and constructs four hydrogen bonds in the binding sites of the inhibitor of nuclear factor kappa-B kinase subunit beta (IKBKB, PDB ID: 4KIK).

### 2.5. Enzymatic Activity Assay In Vitro

**Figure 8:**
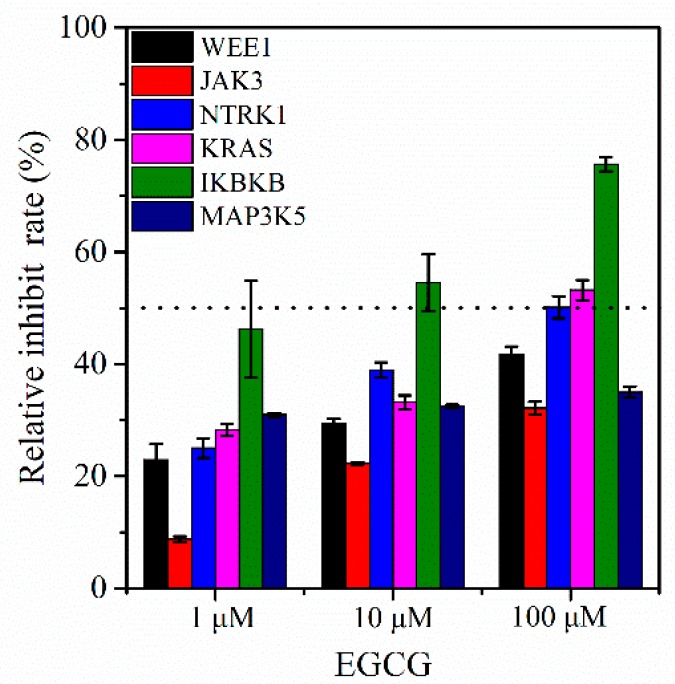
Relative inhibit rate of EGCG potential targets.

To further verify the above-described results, enzymatic activity assays were implemented (Figure 8). We observed that EGCG effectively inhibits IKBKB, KRAS and NTRK1 activity while moderately suppresses WEE1 activity. Furthermore, it only slightly affects the JAK3 and MAP3K5 activity. Taken together, the above-mentioned results suggest that IKBKB, KRAS, NTRK1 and WEE1 might be possible novel potential targets of EGCG.

## 3. Discussion

EGCG, the green tea polyphenol, suppresses tumors through the inhibition of various dynamic tumor-associated proteins and the modulation of relative signaling pathways [4,6,13,14]. Several clinical trials have declared that EGCG is an important anti-tumor compound but very little is known about its potential targets and its molecular mechanisms [12]. Therefore, in the present work, we integrated bioinformatics and computational chemistry methodologies for the discovery of novel EGCG targets and tried to explain its anti-tumor mechanism.

**Figure 9:**
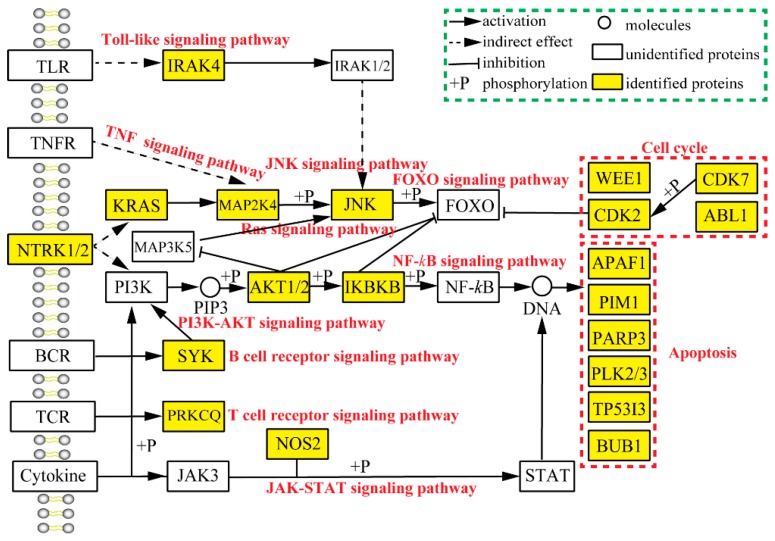
The schematic diagram of EGCG anti-tumor mechanisms based on reverse docking. The black solid arrows, dashed arrows and T-shaped lines represent the activation, indirect effect and inhibition, respectively. The circles, white rectangles and yellow rectangles represent the small molecules, unidentified proteins and identified proteins based on the result of top 40 target proteins through reverse docking. The red word represent the signaling pathway from KEGG terms.

In order to provide a better understanding of the anti-tumor mechanisms of EGCG and its putative target proteins, a schematic diagram was constructed manually based on KEGG pathway maps and related scientific literatures [25], as presented in Figure 9. This diagram explains the anti-tumor mechanisms of EGCG in a very comprehensive way and points out the involvement of 12 signaling pathways and 33 target proteins. Our results also illustrated that EGCG effectively modulates tumor-associated proteins, such as apoptotic proteins (e.g., APAF1, PIM1, PARP3, PLK2/3, TP53I3 and BUB1), cell cycle proteins (e.g., WEE1, CDK2/7 and ABL1), protein kinases (e.g., IRAK4, JNK, JAK3, AKT1/2, PRKCQ, SYK, IKBKB and MAP kinases), receptor proteins (e.g., NTRK1/2), proto-oncogene protein (e.g., KRAS) and synthase protein (e.g., NOS2) [4]. In other words, EGCG alters the target proteins and further regulates relative signaling pathways, including apoptosis, cell cycle, PI3K-AKT, JAK-STAT, JNK, Ras, NF-*k*B, T/B cell receptors, FOXO, Toll-like and TNF signaling pathways.

Previous research illustrated that EGCG induces apoptosis and cell cycle arrest during tumorigenesis through the regulation of NF-*k*B [15,53,54], cyclin D1 [4] and cyclin-dependent kinases (CDK) [4,17]. Similarly, our reverse docking results indicated that EGCG targets CDK4 and CDK7, which are among the top 40 target proteins. Furthermore, Masuda et al. and Aggarwal et al. showed that EGCG inhibits the activation of NF-*k*B in head and neck cancer, breast cancer and colon cancer and it is involved in the NF-*k*B signaling pathway [55,56]. Conversely, we obtained a lower docking score of NFKB1 (nuclear factor NF-kappa B, only –6.2 KJ/mol, Table S2) was detected in our reverse docking research. Interestingly, IKBKB that is involved in NF-*k*B signaling pathway possesses a high docking score and inhibition rate (Table 1 and Figure 8) so based on this information, we can say that IKBKB may be a novel potential target of EGCG. Similarly, Shankar et al. confirmed that EGCG suppressed AKT and ERK phosphorylation and improves the activation of FOXO transcription factors that results in cell cycle arrest and apoptosis [57]. We can easily assume that EGCG targets AKT1/2 due to the high docking scores (Table 1 and Figure 9), whereas FOXO family proteins (FOXO3A and FOXO4) have extremely low docking scores (Table S3). These results demonstrate that EGCG not only modulates the FOXO signaling pathway by the activation of FOXO transcription factors but also regulates the PI3K-AKT signaling pathway through the inhibition of AKT1/2 activity.

Recently, EGCG has been reported to be involved in the inhibition of MAP kinase activity and plays a role in various important signaling pathways, including JNK, RAS and apoptosis [4,16]. Our results indicated that EGCG may suppress JNK1 (MAPK8), MAP3K5 and MAP2K4 according to the docking scores, whereas JNK1 merely has been verified by scientific experiments [46]. Therefore, we also deduced that MAP3K5 and MAP2K4 might be novel potential targets for EGCG. However, the enzymatic activity assay indicated that EGCG can slightly affect MAP3K5 activity. In addition, Senggunprai et al. showed that EGCG suppresses the JAK-STAT signaling pathway activation via modulating the phosphorylation of STAT1 and STAT3 and inhibiting the expression level of iNOS (inducible nitric oxide synthase) and ICAM-1 (intercellular cell adhesion molecule-1) in cholangiocarcinoma cells [58]. Thus, according to our findings, EGCG may regulate the JAK-STAT signaling pathway by targeting NOS2 and JAK3 (Figure 9). However, the enzymatic activity assays indicated that JAK3 was not an ideal target for EGCG (Figure 8). Unluckily, we did not detect the docking scores of STAT2 and STAT3 due to the unavailability of their crystal structure in the PDB database.

In addition, Byun et al. revealed that EGCG modulates Toll-like signaling pathway via suppressing the expression of 67-kDa laminin receptor (67LR) and downregulating TLR2 and TLR4 on dendritic and leukemia cells [19,20]. In the same way, our reverse docking experiments indicated that EGCG also modulated Toll-like signaling pathway via targeting IRAK4 (Figure 9). Therefore, IRAK4 also may be considered as a potential target for EGCG and the enzymatic activity assays further verified this hypothesis. Excitingly, two novel signaling pathways were regulated via SYK and PRKCQ in B and T cell receptor signaling pathway, respectively (Figure 9). The results suggest that EGCG may suppress SYK and PRKCQ to modulate these important pathways.

In conclusion, we applied a series of computational chemistry, bioinformatics and enzymatic activity assay approaches to explore the anti-tumor mechanisms of EGCG and discovered several potential targets and novel pathways. However, most of the proteins had an unknown crystal structure. In addition, the set of anti-tumor targets was relatively small and it only contained 299 tumor-associated proteins in our works. However, Lauro et al. and Chen et al. established a similar set of targets to implement the reverse docking and it also contained only 126 and 150 different proteins, respectively [26,59]. Regrettably, our work had some shortcomings as it only applied a single docking program, Autodock Vina, to perform reverse docking rather than multiple programs. In addition, our work considered only the top 40 targets in the results of reverse docking, but the excluded proteins may also be potential or known targets of EGCG. The MD simulations were performed to assess the binding stability of compounds and the enzymatic activity assays were further carried out to validate the possible targets. However, the simulation times were not long enough. Similarly, the enzymatic activity was measured to validate the six possible targets, but biological assays were not long and deep enough. In short, the present research provides great inspiration and highly encourages the study of the pharmacological mechanism of natural products, identification of therapeutic targets and discovery of novel signaling pathways.

## 4. Materials and Methods

### 4.1. Construction of Anti-tumor Target Proteins Database

The database of anti-tumor target proteins was constructed based on related scientific studies and literature [26,59,60,61] and was also downloaded from the protein databank (PDB). The undefined binding sites and repeated targets were removed from the database of anti-tumor target proteins. Finally, 299 different target proteins were constructed from PDB and installed in the database of anti-tumor targets.

### 4.2. Reverse Virtual Screening

The crystal structures of the targets were prepared based on the following strategies [26]: (1) removal of the water molecules, heteroatoms, ions, co-crystallized ligands and repeated chains using Pymol 1.3.X software; and (2) completing the missing residues using SWISS-MODELING software. The binding site was determined based on the co-crystallized ligand in crystal structures. The geometry center of the co-crystallized ligand was denoted as the active center box and the size_*x*, size_*y*, size_*z* was set to 22.5, 22.5 and 22.5 using the Autodock Vina plugin in Pymol 1.3.X software [62] (Table S4). The target proteins and small molecule epigallocatechin-3-gallate (EGCG) were automatically processed, including adding polar hydrogens and charges, by using the protein and ligand preparation python script in Autodock tools 1.4.5. Reverse virtual screening was performed by using Autodock Vina software and all parameters were adopted as their defaults [38]. Finally, the conformation of each target–EGCG compound was generated and the binding scores were calculated.

The root means square deviation (RMSD) is used to validate the accuracy of the docking program to replicate the ligand conformation observed in the crystal structure. Therefore, a total of 100 original ligands were selected randomly and redocked into the binding pocket of targets as a test set. The VMD 1.9.3 tool was used to calculate the RMSD of ligand heavy atoms between docking and crystal conformation [26,63].

### 4.3. GO Analysis and KEGG Pathway Enrichment

GO (Gene Ontology) enrichment analysis of the interesting targets was carried out using the DAVID 6.8 tools [35]. The KEGG (Kyoto encyclopedia of genes and genomes) enrichment analysis of the objective targets was assigned using the online KOBAS 3.0 tools [64]. The *p*-value was used to identify the significance of the GO terms and KEGG pathways and *p*-value ≤ 0.05 was regarded as being significant and interesting. The ggplot2 package was installed in the R 3.5.2 software for visualizing terms [65].

### 4.4. Pharmacological Network Analysis

The analysis of PPI (protein–protein interaction) was established according to the STRING database [36]. Based on the results of KEGG pathway, the analysis of TP (Target–pathway) was realized by using Cytoscape 3.7.1 software [66]. The NetworkAnalyzer plugin was applied to obtain the parameters of the network topology, such as nodes, degree and edge betweenness [25].

### 4.5. Molecular Dynamic Simulation

The MD (molecular dynamic) simulations were performed using Gromacs 4.6.5 tools [67,68]. Based on the Autodock Vina, the initial configurations of the EGCG-target complex were considered as a confirmation of MD simulation. The topology files of target proteins were obtained by using the pdb2gmx [37,52]. The topology files of EGCG were generated by the antechamber and tleap tools from Ambertools 18 software using the AM1/BCC charge method [69]. The force field of proteins and ligands were Amberff99SB and GAFF (a general Amber force field), respectively [69]. The complex system was preprocessed by the following steps: (1) the TIP3P water molecules solving the system with the dodecahedron box; (2) Na^+^ and Cl^−^ ions neutralizing the system; and (3) the periodic boundary condition with a minimal distance of 1.2 nm [37]. The systems first had their energy minimized using the steepest descent minimization method to limit the energy to 1000 Kcal/mol/nm [69]. Before the formal simulation, the complex system was carried out at both 100 ps NVT and NPT ensemble with 300 K temperature and 1 atm pressure [22,51]. The long-range electrostatic interactions were calculated using the PME (Particle Mesh Ewald) method and the hydrogen bonds were constrained using the LINCS (Linear Constraint Solver) algorithm. The non-bonded cutoff distance was set to 12 Å and the SETTLE algorithm was applied to restrict water molecules. The 20 ns MD simulations were performed with a time step of 2 fs and the atomic coordinates were recorded every 5000 fs. Pymol 1.3.X was further used to generate visual conformation.

### 4.6. Enzymatic Activity Assay In Vitro

EGCG (HPLC ≥ 99%) was purchased from Alfa biotechnology corporation (Chengdu, China). The kinase kits of six interesting targets were obtained from Mlbio biotechnology corporation (Shanghai, China). The assays were carried out according to the instructions of the kinase kits. Firstly, 50 μL of the compound solution was added to 96-well plates and incubated for 30 min at 37 °C. Second, the compound solution was removed and 50 μL of the HRP-conjugate reagent was added and incubated for 30 min at 37 °C. Third, HRP-conjugate reagent was removed before chromogen solution A and B was added and incubated for 10 min at 37 °C. Finally, the 50 μL stop solution was added and Bio-Rad iMark microplate reader was used to measure the activity at 450 nm.

## 5. Conclusions

We integrated the computational chemistry and bioinformatics techniques to analyze the anti-tumor mechanism of EGCG and explore possible targets and pathways. According to our findings the mechanism involves 12 signaling transduction pathways, such as apoptosis, PI3K-AKT, MAPK, FOXO and KRAS, and 33 vital target proteins. Furthermore, we also discovered that six tumor-associated proteins may be novel potential targets for EGCG but four targets were only moderately inhibited, including IKBKB, KRAS, WEE1 and NTRK1. Moreover, the establishment of networks between tumor-related proteins and EGCG may have important implications for understanding the underlying anti-tumor mechanisms of EGCG. Convincingly, a new therapeutic strategy for tumor may be developed from the identified targets and pathways. In summary, this study provided important information for studying the pharmacological mechanism of EGCG and identifying novel potential therapeutic targets.

## Figures and Tables

**Table 1:** The docking score of top 40 target proteins based on reverse docking.

PDB_ID	Symbol	Uniprot_ID	Ligand_ID	Lig_Score	EGCG_Score	Experiment
3UYT	CSNK1D	P48730	0CK	−9.6	−9.2	No
4B6L	PLK3	Q9H4B4	9ZP	−8.2	−9.2	No
4K4E	TNKS	O95271	4KE	−9.2	−9.2	No
3MVH	AKT1	P31749	WFE	−1.8	−9.1	Yes, [1,45]
2CLQ	MAP3K5	Q99683	STU	−12.0	−9.1	No
3GFT	KRAS	P01116	GNP	−7.6	−9.1	No
4TVJ	PARP2	Q9UGN5	09L	−12.5	−10.8	No
2DQ7	FYN	P06241	STU	−12.1	−10.0	Yes, [39]
4KIK	IKBKB	O14920	KSA	−14.0	−10.0	No
3EQR	TNK2	Q07912	T74	−10.2	−9.9	No
3MTF	ACVR1	Q04771	A3F	−9.3	−9.9	No
4GV0	PARP3	Q9Y6F1	8ME	−9.4	−9.9	No
1M6I	AIFM1	O95831	FAD	−7.4	−9.8	No
1UA2	CDK7	P50613	ATP	−8.0	−9.8	No
1Z6T	APAF1	O14727	ADP	−8.1	−9.8	Yes, [48]
1ZXM	TOP2A	P11388	ANP	−8.2	−9.8	No
2WQN	NEK7	Q8TDX7	ADP	−8.0	−9.6	No
4NOS	NOS2	P35228	H2B	−4.0	−9.6	Yes, [40,41]
4R8Q	BUB1	O43683	ADP	−7.1	−9.6	No
1YVJ	JAK3	P52333	4ST	−11.9	−9.5	No
2IW9	CDK2	P24941	4SP	−7.2	−9.5	Yes, [42,43]
2V7A	ABL1	P00519	627	−7.6	−9.5	Yes, [44]
4I5M	PLK2	Q9NYY3	R78	−8.4	−9.50	No
6CIC	NOS1	P29475	7R2	−8.8	−9.50	No
1XBC	SYK	P43405	STU	−12.5	−9.4	Yes, [8]
2JDR	AKT2	P31751	L20	−10.2	−9.4	Yes, [1,45]
2JED	PRKCQ	Q04759	LG8	−10.8	−9.4	No
3ALN	MAP2K4	P45985	ANP	−7.9	−9.4	No
3ELJ	MAPK8	P45983	GS7	−8.0	−9.4	Yes, [46]
3NR9	CLK2	P49760	NR9	−8.7	−9.4	No
4IFC	PRPF4B	Q13523	ADP	−8.0	−9.4	No
1X8B	WEE1	P30291	824	−11.6	−9.3	No
2J8Z	TP53I3	Q53FA7	NAP	−7.8	−9.3	No
2NRU	IRAK4	Q9NWZ3	T12	−8.4	−9.3	Yes, [47]
3OP5	VRK1	Q99986	REB	−2.1	−9.3	No
4AOJ	NTRK1	P04629	V4Z	−8.8	−9.3	No
5DN3	AURKA	O14965	5DN	−9.5	−9.3	No
5YQX	BRD4	O60885	E0K	−7.6	−9.3	No
2XIK	STK25	O00506	J60	−1.9	−9.2	No
3JYA	PIM1	P11309	LWG	−8.3	−9.2	No

Note: Lig_score and EGCG_score represented the docking scores of original ligand and EGCG, respectively. Uniprot_ID and Ligand_ID represented the identification (ID) of the target proteins from uniport database and original ligand from PDB database, respectively.

## Supplementary Materials

The following are available online. Table S1: The top15 terms for genetic association database. Table S2: The results of the reverse docking for AutoDock Vina. Table S3: The results of KEGG pathway enrichment. Table S4: The center box of anti-tumor targets for AutoDock Vina.

## Abbreviation

EC	Epicatechin
GC	Gallocatechin
EGC	Epigallocatechin
CG	Catechin-3-gallate
ECG	Epicatechin-3-gallate
GCG	Gallocatechin-3-gallate
EGCG	Epigallocatechin-3-gallate
Pin1	Peptidyl-prolyl *cis/trans* isomerase NIMA-interacting 1
MD	Molecular Dynamic
GO	Gene Ontology
KEGG	Kyoto Encyclopedia of Genes and Genomes
DAVID	The Database for Annotation, Visualization and Integrated Discovery
PPI	Protein–protein Interaction
TP	Target–pathway
NVT	Constant number of particles, volume and temperature
NPT	Constant number of particles, pressure and temperature
PME	Particle Mesh Ewald
LINCS	Linear Constraint Solver
RMSD	Root Mean Square Deviation
GAD	Genetic Association Database
FYN	Proto-oncogene tyrosine-protein kinase FYN
NOS2	Nitric Oxide Synthase 2
CDK2	Cyclin-Dependent Kinase 2
CDK7	Cyclin-Dependent Kinase 7
ABL1	Abelson Murine Leukemia Viral Oncogene Homolog 1
SYK	Spleen tyrosine kinase
AKT2	Serine-threonine protein kinase AKT2
MAPK8	Mitogen-activated protein kinase 8
AKT1	Serine-threonine protein kinase AKT1
KRAS	Kirsten Rat Sarcoma Viral Oncogene
IRAK4	Interleukin-1 Receptor-Associated Kinase 4
WEE1	Mitosis inhibitor protein kinase WEE1
JAK3	Tyrosine-protein kinase JAK3
APAF1	Apoptotic Protease Activating Factor 1
NTRK1	High affinity nerve growth factor receptor 1
NTRK2	High affinity nerve growth factor receptor 2
MAP3K5	Mitogen-Activated Protein Kinase Kinase Kinase 5
IKBKB	Inhibitor of nuclear factor kappa-B kinase subunit beta
TP53I3	Quinone oxidoreductase PIG3
PIM1	Serine/threonine-protein kinase PIM1
PARP3	Poly ADP-ribose polymerase 3
PLK2	Serine/threonine-protein kinase PLK2
PLK3	Serine/threonine-protein kinase PLK3
BUB1	Mitotic checkpoint serine/threonine-protein kinase BUB1
AKT1	RAC-alpha serine/threonine-protein kinase 1
AKT2	RAC-alpha serine/threonine-protein kinase 2
PRKCQ	Protein kinase C theta type
JAK	Tyrosine-protein kinase JAK
STAT	Signal transducer and activator of transcription
NF-*k*B	Nuclear factor NF-kappa-B
FOXO	Forkhead Box O
PI3K	Phosphoinositide 3-kinase
iNOS	Inducible nitric oxide synthase
ICAM-1	Intercellular cell adhesion molecule-1
TNF	Tumor Necrosis Factor
TNFR	Tumor necrosis factor receptor
TLR	Toll-like receptors
BCR	B cell receptor
TCR	T cell receptor
NFKB1	Nuclear factor NF-kappa-B
MAPK	Mitogen-activated protein kinase

## References

[1] Park S.Y., Lee Y.-K., Kim Y.-M., Park O.J., Shin J.-I. (2013). Control of AMP-activated protein kinase, Akt and mTOR in EGCG-treated HT-29 colon cancer cells. Food Sci. Biotechnol..

[2] Wang Y.-Q., Lu J.-L., Liang Y.-R., Li Q.-S. (2018). Suppressive effects of egcg on cervical cancer. Molecules.

[3] Benvenuto M., Fantini M., Masuelli L., De Smaele E., Zazzeroni F., Tresoldi I., Calabrese G., Galvano F., Modesti A., Bei R. (2013). Inhibition of ErbB receptors, Hedgehog and NF-kappaB signaling by polyphenols in cancer. Front. Biosci..

[4] Singh B.N., Shankar S., Srivastava R.K. (2011). Green tea catechin, epigallocatechin-3-gallate (EGCG): Mechanisms, perspectives and clinical applications. Biochem. Pharmacol..

[5] Gao Z., Han Y., Hu Y., Wu X., Wang Y., Zhang X., Fu J., Zou X., Zhang J., Chen X. (2016). Targeting HO-1 by epigallocatechin-3-gallate reduces contrast-induced renal injury via anti-oxidative stress and anti-inflammation pathways. PLoS ONE.

[6] Riegsecker S., Wiczynski D., Kaplan M.J., Ahmed S. (2013). Potential benefits of green tea polyphenol EGCG in the prevention and treatment of vascular inflammation in rheumatoid arthritis. Life Sci..

[7] Tedeschi E., Suzuki H., Menegazzi M. (2002). Antiinflammatory action of EGCG, the main component of green tea, through STAT-1 inhibition. Ann. N. Y. Acad. Sci..

[8] Deana R., Turetta L., Donella-Deana A., Donà M., Brunati A.M., De Michiel L., Garbisa S. (2003). Green tea epigallocatechin-3-gallate inhibits platelet signalling pathways triggered by both proteolytic and non-proteolytic agonists. Thromb. Haemost..

[9] Rashidi B., Malekzadeh M., Goodarzi M., Masoudifar A., Mirzaei H. (2017). Green tea and its anti-angiogenesis effects. Biomed. Pharmacother..

[10] García-Vilas J.A., Quesada A.R., Medina M.Á. (2016). Screening of synergistic interactions of epigallocatechin-3-gallate with antiangiogenic and antitumor compounds. Synergy.

[11] Diniz C., Suliburska J., Ferreira I.M. (2017). New insights into the antiangiogenic and proangiogenic properties of dietary polyphenols. Mol. Nutr. Food Res..

[12] Zheng R., Chen T.-S., Lu T. (2011). A comparative reverse docking strategy to identify potential antineoplastic targets of tea functional components and binding mode. Int. J. Mol. Sci..

[13] Khan N., Afaq F., Saleem M., Ahmad N., Mukhtar H. (2006). Targeting multiple signaling pathways by green tea polyphenol (−)-epigallocatechin-3-gallate. Cancer Res..

[14] Saeki K., Hayakawa S., Nakano S., Ito S., Oishi Y., Suzuki Y., Isemura M. (2018). In vitro and in silico studies of the molecular interactions of epigallocatechin-3-o-gallate (egcg) with proteins that explain the health benefits of green tea. Molecules.

[15] Gupta S., Hastak K., Afaq F., Ahmad N., Mukhtar H. (2004). Essential role of caspases in epigallocatechin-3-gallate-mediated inhibition of nuclear factor kappaB and induction of apoptosis. Oncogene.

[16] Shankar S., Suthakar G., Srivastava R.K. (2007). Epigallocatechin-3-gallate inhibits cell cycle and induces apoptosis in pancreatic cancer. Front. Biosci..

[17] Masuda M., Suzui M., Weinstein I.B. (2001). Effects of epigallocatechin-3-gallate on growth, epidermal growth factor receptor signaling pathways, gene expression and chemosensitivity in human head and neck squamous cell carcinoma cell lines. Clin. Cancer Res..

[18] Bartholome A., Kampkötter A., Tanner S., Sies H., Klotz L.-O. (2010). Epigallocatechin gallate-induced modulation of FoxO signaling in mammalian cells and C. elegans: FoxO stimulation is masked via PI3K/Akt activation by hydrogen peroxide formed in cell culture. Arch. Biochem. Biophys..

[19] Byun E.H., Fujimura Y., Yamada K., Tachibana H. (2010). TLR4 signaling inhibitory pathway induced by green tea polyphenol epigallocatechin-3-gallate through 67-kDa laminin receptor. J. Immunol..

[20] Byun E.-H., Omura T., Yamada K., Tachibana H. (2011). Green tea polyphenol epigallocatechin-3-gallate inhibits TLR2 signaling induced by peptidoglycan through the polyphenol sensing molecule 67-kDa laminin receptor. FEBS Lett..

[21] Urusova D.V., Shim J.-H., Kim D.J., Jung S.K., Zykova T.A., Carper A., Bode A.M., Dong Z. (2011). Epigallocatechin-gallate suppresses tumorigenesis by directly targeting Pin1. Cancer Prev. Res..

[22] Xi L., Wang Y., He Q., Zhang Q., Du L. (2016). Interaction between Pin1 and its natural product inhibitor epigallocatechin-3-gallate by spectroscopy and molecular dynamics simulations. Spectrochim. Acta Part A Mol. Biomol. Spectrosc..

[23] Nakano S., Megro S.-I., Hase T., Suzuki T., Isemura M., Nakamura Y., Ito S. (2018). Computational molecular docking and X-ray crystallographic studies of catechins in new drug design strategies. Molecules.

[24] Negri A., Naponelli V., Rizzi F., Bettuzzi S. (2018). Molecular Targets of Epigallocatechin—Gallate (EGCG): A Special Focus on Signal Transduction and Cancer. Nutrients.

[25] Li H., Sun X., Yu F., Xu L., Miu J., Xiao P. (2018). In silico Investigation of the Pharmacological Mechanisms of Beneficial Effects of *Ginkgo biloba* L. on Alzheimer’s Disease. Nutrients.

[26] Chen F., Wang Z., Wang C., Xu Q., Liang J., Xu X., Yang J., Wang C., Jiang T., Yu R. (2017). Application of reverse docking for target prediction of marine compounds with anti-tumor activity. J. Mol. Graph. Model..

[27] Kharkar P.S., Warrier S., Gaud R.S. (2014). Reverse docking: A powerful tool for drug repositioning and drug rescue. Future Med. Chem..

[28] Huang H., Zhang G., Zhou Y., Lin C., Chen S., Lin Y., Mai S., Huang Z. (2018). Reverse screening methods to search for the protein targets of chemopreventive compounds. Front. Chem..

[29] Ma C., Tang K., Liu Q., Zhu R., Cao Z. (2013). Calmodulin as a Potential Target by Which Berberine Induces Cell Cycle Arrest in Human Hepatoma B el7402 Cells. Chem. Biol. Drug Des..

[30] Liu X., Ouyang S., Yu B., Liu Y., Huang K., Gong J., Zheng S., Li Z., Li H., Jiang H. (2010). PharmMapper server: A web server for potential drug target identification using pharmacophore mapping approach. Nucleic Acids Res..

[31] Zhang S., Lu W., Liu X., Diao Y., Bai F., Wang L., Shan L., Huang J., Li H., Zhang W. (2011). Fast and effective identification of the bioactive compounds and their targets from medicinal plants via computational chemical biology approach. MedChemComm.

[32] Wang X., Zhang X., Xia P., Zhang J., Wang Y., Zhang R., Giesy J.P., Shi W., Yu H. (2017). A high-throughput, computational system to predict if environmental contaminants can bind to human nuclear receptors. Sci. Total Environ..

[33] Park K., Cho A.E. (2017). Using reverse docking to identify potential targets for ginsenosides. J. Ginseng Res..

[34] Kanehisa M., Goto S. (2000). KEGG: Kyoto encyclopedia of genes and genomes. Nucleic Acids Res..

[35] Huang D.W., Sherman B.T., Lempicki R.A. (2008). Systematic and integrative analysis of large gene lists using DAVID bioinformatics resources. Nature Protoc..

[36] Franceschini A., Szklarczyk D., Frankild S., Kuhn M., Simonovic M., Roth A., Lin J., Minguez P., Bork P., Von Mering C. (2012). STRING v9. 1: Protein–protein interaction networks, with increased coverage and integration. Nucleic Acids Res..

[37] Li J., Zhou N., Luo K., Zhang W., Li X., Wu C., Bao J. (2014). In silico discovery of potential VEGFR-2 inhibitors from natural derivatives for anti-angiogenesis therapy. Int. J. Mol. Sci..

[38] Trott O., Olson A.J. (2010). AutoDock Vina: Improving the speed and accuracy of docking with a new scoring function, efficient optimization and multithreading. J. Comput. Chem..

[39] He Z., Tang F., Ermakova S., Li M., Zhao Q., Cho Y.Y., Ma W.Y., Choi H.S., Bode A.M., Yang C.S. (2008). Fyn is a novel target of (−)-epigallocatechin gallate in the inhibition of JB6 Cl41 cell transformation. Mol. Carcinog..

[40] Ahmed S., Rahman A., Hasnain A., Lalonde M., Goldberg V., Haqqi T. (2002). Green tea polyphenol EGCG inhibits the IL-1 beta-induced activity an d expression of COX-2 and NOS-2 in human chondrocytes. Free Radic. Biol. Med..

[41] Oliva J., Bardag-Gorce F., Tillman B., French S.W. (2011). Protective effect of quercetin, EGCG, catechin and betaine against oxidative stress induced by ethanol in vitro. Exp. Mol. Pathol..

[42] Hung P.-F., Wu B.-T., Chen H.-C., Chen Y.-H., Chen C.-L., Wu M.-H., Liu H.-C., Lee M.-J., Kao Y.-H. (2005). The antimitogenic effect of green tea (−)-epigallocatechin gallate on 3T3-L1 preadipocytes depends on the Erk and Cdk2 pathways. Am. J. Physiol. Cell Physiol..

[43] Wu B.-T., Hung P.-F., Chen H.-C., Huang R.-N., Chang H.-H., Kao Y.-H. (2005). The apoptotic effect of green tea (−)-epigallocatechin gallate on 3T3-L1 preadipocytes depends on the Cdk2 pathway. J. Agric. Food Chem..

[44] Chen H., Adams E., Van Schepdael A. (2013). Study of Abl1 tyrosine kinase inhibitors by liquid chromatography–electrospray ionization-mass spectrometry. Talanta.

[45] Van Aller G.S., Carson J.D., Tang W., Peng H., Zhao L., Copeland R.A., Tummino P.J., Luo L. (2011). Epigallocatechin gallate (EGCG), a major component of green tea, is a dual phosphoinositide-3-kinase/mTOR inhibitor. Biochem. Biophys. Res. Commun..

[46] Park S.B., Bae J.W., Kim J.M., Lee S.G., Han M. (2012). Antiproliferative and apoptotic effect of epigallocatechin-3-gallate on Ishikawa cells is accompanied by sex steroid receptor downregulation. Int. J. Mol. Med..

[47] Singh A.K., Umar S., Riegsecker S., Chourasia M., Ahmed S. (2016). Regulation of Transforming Growth Factor β–Activated Kinase Activation by Epigallocatechin-3-Gallate in Rheumatoid Arthritis Synovial Fibroblasts: Suppression of K63-Linked Autoubiquitination of Tumor Necrosis Factor Receptor–Associated Factor 6. Arthritis Rheumatol..

[48] Wu P.-P., Kuo S.-C., Huang W.-W., Yang J.-S., Lai K.-C., Chen H.-J., Lin K.-L., Chiu Y.-J., Huang L.-J., Chung J.-G. (2009). (–)-Epigallocatechin gallate induced apoptosis in human adrenal cancer NCI-H295 cells through caspase-dependent and caspase-independent pathway. Anticancer Res..

[49] Wang W., Wu J., Zhang Q., Li X., Zhu X., Wang Q., Cao S., Du L. (2019). Mitochondria-mediated apoptosis was induced by oleuropein in H1299 cells involving activation of p38 MAP kinase. J. Cell. Biochem..

[50] Li X., Sun R., Chen W., Lu B., Li X., Wang Z., Bao J. (2014). A systematic in silico mining of the mechanistic implications and therapeutic potentials of estrogen receptor (ER)-α in breast cancer. PLoS ONE.

[51] Wang W., Li X., Wang Q., Zhu X., Zhang Q., Du L. (2018). The acidic pH-induced structural changes in apo-CP43 by spectral methodologies and molecular dynamics simulations. J. Mol. Struct..

[52] Zhao M.-L., Wang W., Nie H., Cao S.-S., Du L.-F. (2018). In silico structure prediction and inhibition mechanism studies of AtHDA14 as revealed by homology modeling, docking, molecular dynamics simulation. Comput. Biol. Chem..

[53] Yang F., Oz H.S., Barve S., De Villiers W.J., McClain C.J., Varilek G.W. (2001). The green tea polyphenol (−)-epigallocatechin-3-gallate blocks nuclear factor-κB activation by inhibiting IκB kinase activity in the intestinal epithelial cell line IEC-6. Mol. Pharmacol..

[54] Oz H.S., Ebersole J.L. (2010). Green tea polyphenols mediated apoptosis in intestinal epithelial cells by a FADD-dependent pathway. J. Cancer Ther..

[55] Masuda M., Suzui M., Lim J.T., Weinstein I.B. (2003). Epigallocatechin-3-gallate inhibits activation of HER-2/neu and downstream signaling pathways in human head and neck and breast carcinoma cells. Clin. Cancer Res..

[56] Aggarwal B.B., Shishodia S. (2006). Molecular targets of dietary agents for prevention and therapy of cancer. Biochem. Pharmacol..

[57] Shankar S., Marsh L., Srivastava R.K. (2013). EGCG inhibits growth of human pancreatic tumors orthotopically implanted in Balb C nude mice through modulation of FKHRL1/FOXO3a and neuropilin. Mol. Cell. Biochem..

[58] Senggunprai L., Kukongviriyapan V., Prawan A., Kukongviriyapan U. (2014). Quercetin and EGCG exhibit chemopreventive effects in cholangiocarcinoma cells via suppression of JAK/STAT signaling pathway. Phytother. Res..

[59] Lauro G., Romano A., Riccio R., Bifulco G. (2011). Inverse virtual screening of antitumor targets: Pilot study on a small database of natural bioactive compounds. J. Nat. Prod..

[60] Verma N., Rai A.K., Kaushik V., Brünnert D., Chahar K.R., Pandey J., Goyal P. (2016). Identification of gefitinib off-targets using a structure-based systems biology approach; their validation with reverse docking and retrospective data mining. Sci. Rep..

[61] Zahler S., Tietze S., Totzke F., Kubbutat M., Meijer L., Vollmar A.M., Apostolakis J. (2007). Inverse in silico screening for identification of kinase inhibitor targets. Chem. Biol..

[62] Seeliger D., de Groot B.L. (2010). Ligand docking and binding site analysis with PyMOL and Autodock/Vina. J. Comput. Aided Mol. Des..

[63] Humphrey W., Dalke A., Schulten K. (1996). VMD: Visual molecular dynamics. J. Mol. Graph..

[64] Xie C., Mao X., Huang J., Ding Y., Wu J., Dong S., Kong L., Gao G., Li C.-Y., Wei L. (2011). KOBAS 2.0: A web server for annotation and identification of enriched pathways and diseases. Nucleic Acids Res..

[65] Wickham H. (2016). ggplot2: Elegant Graphics for Data Analysis.

[66] Shannon P., Markiel A., Ozier O., Baliga N.S., Wang J.T., Ramage D., Amin N., Schwikowski B., Ideker T. (2003). Cytoscape: A software environment for integrated models of biomolecular interaction networks. Genome Res..

[67] Hess B., Kutzner C., Van Der Spoel D., Lindahl E. (2008). GROMACS 4: Algorithms for highly efficient, load-balanced and scalable molecular simulation. J. Chem. Theory Comput..

[68] Van Der Spoel D., Lindahl E., Hess B., Groenhof G., Mark A.E., Berendsen H.J. (2005). GROMACS: Fast, flexible and free. J. Comput. Chem..

[69] Wang X., Lu K., Luo H., Liang D., Long X., Yuan Y., Wu C., Bao J. (2018). In silico identification of small molecules as novel LXR agonists for the treatment of cardiovascular disease and cancer. J. Mol. Model..

