# Supplementary material for: In Silico Investigation of the Anti-Tumor Mechanisms of Epigallocatechin-3-Gallate
Source: Molecules. 2019 Apr 11;24(7):1445. doi: 10.3390/molecules24071445 (PMC6480119; doi:10.3390/molecules24071445)
Supplement: Supplementary file 1 [file molecules-24-01445-s001.pdf]

Supplement materials

Table S1. The top15 terms for genetic association database.

Term	Count	Percentage %	*p*-Value	List	FDR
Lung Cancer	73	25.34	6.70E-36	271	1.16E-32
Breast Cancer	67	23.26	2.92E-30	271	5.06E-27
Bladder Cancer	60	20.83	3.33E-27	271	5.78E-24
Esophageal adenocarcinoma	52	18.06	6.24E-29	271	1.08E-25
Colorectal Cancer	51	17.71	4.99E-24	271	8.66E-21
Prostate cancer	42	14.58	7.12E-14	271	1.23E-10
Ovarian cancer	39	13.54	2.04E-16	271	3.89E-13
Pancreatic Neoplasms	20	6.94	1.45E-08	271	2.52E-05
Thyroid cancer	19	6.59	9.98E-11	271	1.73E-07
Rheumatoid arthritis	19	6.60	8.82E-08	271	1.53E-04
Neoplasms	17	5.90	4.58E-08	271	7.94E-05
Stomach cancer	16	5.56	1.17E-09	271	2.03E-06
Head and neck cancer	15	5.21	5.23E-09	271	9.07E-06
Stomach Neoplasms	14	4.86	6.20E-07	271	1.08E-3
Leukemia	14	4.86	1.02E-06	271	1.78 E-3

Table S2. The results of the reverse docking for AutoDock Vina.

PDB	Protein	Gene_Name	Uniprot_ID	Score
4TVJ	Poly [ADP-ribose] polymerase 2	PARP2	Q9UGN5	-10.80
2DQ7	Proto-oncogene tyrosine-protein kinase Fyn	FYN	P06241	-10.00
4KIK	Inhibitor of nuclear factor kappa-B kinase subunit beta	IKBKB	O14920	-10.00
3EQR	Activated CDC42 kinase 1	ACK1/TNK2	Q07912	-9.90
3MTF	Activin receptor type-1	ACVR1	Q04771	-9.90
4GV0	Poly [ADP-ribose] polymerase 3	PARP3	Q9Y6F1	-9.90
1M6I	Programmed cell death protein 8	AIF	O95831	-9.80
1UA2	Cell division protein kinase 7	CDK7	P50613	-9.80
1Z6T	Apoptotic protease activating factor 1	APAF1	O14727	-9.80
1ZXM	DNA topoisomerase II	TOPII	P11388	-9.80
2WOU	Serine/Threonine-protein kinase NEK7	NEK7	Q8TDX7	-9.60
4NOS	Inducible nitric oxide synthase	NOS2	P35228	-9.60
4R8Q	Mitotic checkpoint serine/threonine-protein kinase BUB1	BUB1	O43683	-9.60
1YVJ	Tyrosine-protein kinase JAK3	JAK3	P52333	-9.50
2IW9	Cell division protein kinase 2	CDK2	P24941	-9.50
2V7A	Tyrosine-protein kinase ABL1	ABL1	P00519	-9.50
4I5M	Serine/threonine-protein kinase PLK2	PLK2	Q9NYY3	-9.50
6CIC	Interleukin-1 receptor-associated kinase 1	IRAK1	P51617	-9.50
1XBC	Tyrosine-protein kinase SYK	SYK	P43405	-9.40
2JDR	RAC-beta serine/threonine-protein kinase	AKT2	P31751	-9.40
2JED	Protein kinase C theta	PRKCQ	Q04759	-9.40
3ALN	Dual specificity mitogen-activated protein kinase kinase 4	MAP2K4	P45985	-9.40
3ELJ	Mitogen-activated protein kinase 8	JNK1	P45983	-9.40
3NR9	Dual specificity protein kinase CLK2	CLK2	P49760	-9.40
4IFC	Serine/threonine-protein kinase PRP4 homolog	PRPF4B	Q13523	-9.40
1X8B	Wee1-like protein kinase	WEE1	P30291	-9.30
2J8Z	Quinone oxidoreductase	PIG3	Q53FA7	-9.30
2NRU	Interleukin-1 receptor-associated kinase 4	IRAK4	Q9NWZ3	-9.30
3OP5	Serine/threonine-protein kinase VRK1	VRK1	Q99986	-9.30
4AOJ	High affinity nerve growth factor receptor	NTRK1	P04629	-9.30
5DN3	Aurora kinase A	AURKA	O14965	-9.30
5YQX	Bromodomain-containing protein 4	BRD4	O60885	-9.30
2XIK	Serine/threonine protein kinase 25	STK25	O00506	-9.20
3JYA	Serine/threonine-protein kinase Pim1	PIM1	P11309	-9.20
3UYT	Casein kinase I isoform delta	CSNK1D	P48730	-9.20
4B6L	Serine/threonine-protein kinase PLK3	PLK3	Q9H4B4	-9.20
4K4E	Tankyrase-1	TNKS1	O95271	-9.20
1YW9	Methionine aminopeptidase 2	METAP2	P50579	-9.10

2CLQ	Mitogen-activated protein kinase kinase kinase kinase 5	MAP3K5	Q99683	-9.10
2IVT	Tyrosine-protein kinase receptir RET precursor	RET	P07949	-9.10
3BHH	Calcium/calmodulin-dependent protein kinase type II subunit beta	CamKIIB	Q13554	-9.10
3CBL	Tyrosine-protein kinase Fes/Fps	FES	P07332	-9.10
3MVH	RAC-alpha serine/threonine-protein kinase	AKT1	P31749	-9.10
4MQ1	Dual specificity tyrosine-phosphorylation-regulated kinase 1A	DYRK1A	Q13627	-9.10
4OTP	Serine/threonine-protein kinase RIO1	RIOK1	Q9BRS2	-9.10
5FBO	Histone deacetylase 8	HDAC8	Q9BY41	-9.10
6FYV	Dual specificity protein kinase CLK4	CLK4	Q9HAZ1	-9.10
1AD5	Tyrosine-protein kinase HCK	HCK	P08631	-9.00
2JAM	Calcium/calmodulin-dependent protein kinase type 1G	CAMK1G	Q96NX5	-9.00
2R4B	Receptor tyrosine-protein kinase erbB-4	ERBB4	Q15303	-9.00
3GFT	GTPase Kras	KRAS	P01116	-9.00
4G31	Eukaryotic translation initiation factor 2-alpha kinase 3	EIF2AK3	Q9NZJ5	-9.00
4OEL	Cathepsin C	CTSC	P53634	-9.00
2VX1	Ephrin type-B receptor 4	EphB4	P54760	-8.90
2XXZ	Lysine-specific demethylase 6B	KDM6B	O15054	-8.90
3A60	Ribosomal protein S6 kinase beta-1	RPS6KB1	P23443	-8.90
3C0G	Peripheral plasma membrane protein CASK	CASK	O14936	-8.90
3E64	Tyrosine-protein kinase JAK2	JAK2	O60674	-8.90
3IW4	Protein kinase C alpha	PRKCA	P17252	-8.90
3JXU	Heat shock 70 kDa protein 1	HSPA1A	P0DMV8	-8.90
3QFV	Serine/threonine-protein kinase MRCK beta	CDC42BPB	Q9Y5S2	-8.90
4TNB	G protein-coupled receptor kinase 5	GRK5	P34947	-8.90
5YR4	Methionine aminopeptidase 1	METAP1	P53582	-8.90
1U59	Tyrosine-protein kinase ZAP-70	ZAP70	P43403	-8.80
2I0E	Protein kinase C beta	PRKCB	P05771	-8.80
2WGJ	Heat shock protein, HSP90-alpha	HSP90AA1	P07900	-8.80
2WU6	Dual specificity protein kinase CLK3	CLK3	P49761	-8.80
3KRW	Beta-adrenergic receptor kinase 1	GRK2	P25098	-8.80
3PJ1	Tyrosine-protein kinase BTK	BTK	Q06187	-8.80
1AGW	FGF receptor 1	FGFR1	P11362	-8.70
1HVY	Thymidylate synthase	TYMS	P04818	-8.70
2OJG	Mitogen-activated protein kinase 1	MAPK1	P28482	-8.70
3AMB	cAMP-dependent protein kinase catalytic subunit alpha	PKACA	P17612	-8.70
3C4C	Serine/threonine-protein kinase B-raf	BRAF	P15056	-8.70
3DV3	Dual specificity mitogen-activated protein	MEK1	Q02750	-8.70

	kinase kinase 1			
3F7Z	Glycogen synthase kinase-3 beta	GSK3B	P49841	-8.70
3HIG	Amiloride-sensitive amine oxidase	AOC1	P19801	-8.70
3KN5	Ribosomal protein S6 kinase alpha-5	RPS6KA5	O75582	-8.70
3MDY	Peptidyl-prolyl cis-trans isomerase FKBP1A	FKBP1A	P62942	-8.70
3P23	Serine/threonine-protein kinase/endoribonuclease IRE1	ERN1	O75460	-8.70
4AT3	BDNF/NT-3 growth factors receptor	NTRK2	Q16620	-8.70
4WSQ	AP2-associated protein kinase 1	AAK1	Q2M2I8	-8.70
5D9K	Ribosomal protein S6 kinase alpha-3	RPS6KA3	P51812	-8.70
2QRV	DNA (cytosine-5)-methyltransferase 3-like	DNMT3L	Q9UJW3	-8.60
3FZH	Heat shock cognate 71 kDa protein	HSPA8	P11142	-8.60
3T9T	Tyrosine-protein kinase ITK/TSK	ITK	Q08881	-8.60
3V8S	Rho-associated protein kinase 1	ROCK1	Q13464	-8.60
4AF3	Aurora kinase B	AURKB	Q96GD4	-8.60
4IM0	Serine/threonine-protein kinase TBK1	TBK1	Q9UHD2	-8.60
4YHJ	G protein-coupled receptor kinase 4	GRK4	P32298	-8.60
5C8W	cGMP-dependent protein kinase 2	PRKG2	Q13237	-8.60
5UKJ	Phosphatidylinositol 4,5-bisphosphate 3-kinase catalytic subunit alpha isoform	PIK3CA	P42336	-8.60
5XY1	Tyrosine-protein kinase Lyn	LYN	P07948	-8.60
2A2R	Glutathione S-transferase P	GSTP1	P09211	-8.50
2J6M	Epidermal growth factor receptor	EGFR	P00533	-8.50
2X7G	SRSF protein kinase 2	SRPK2	P78362	-8.50
2Z7Q	Ribosomal protein S6 kinase alpha-1	RPS6KA1	Q15418	-8.50
3A7H	Serine/threonine-protein kinase 24	STK24	Q9Y6E0	-8.50
3EYG	Tyrosine-protein kinase JAK1	JAK1	P23458	-8.50
3GDQ	Heat shock 70 kDa protein 1-like	HSPA1L	P34931	-8.50
3HNA	Histone-lysine N-methyltransferase	EHMT1	Q9H9B1	-8.50
3PLS	Macrophage-stimulating protein receptor	MST1R	Q04912	-8.50
3ZNR	Histone deacetylase 7	HDAC7	Q8WUI4	-8.50
4BC6	Serine/threonine protein kinase 10	STK10	O94804	-8.50
5E1S	Insulin receptor	INSR	P06213	-8.50
5LHD	Aminopeptidase N	ANPEP	P15144	-8.50
6AX4	B-lymphocyte antigen CD19	CD19	P15391	-8.50
2VD5	DMPK protein	DMPK	Q09013	-8.40
2WI6	TGF-beta receptor type-1	TGFBR1	P36897	-8.40
2XKF	Serine/Threonine-protein kinase NEK2	NEK2	P51955	-8.40
2ZV2	Calcium/calmodulin-dependent protein kinase kinase 2	CAMKK2	Q96RR4	-8.40
3BIY	Histone acetyltransferase p300	EP300	Q09472	-8.40
3BLQ	Cell division protein kinase 9	CDK9	P50750	-8.40
3LDP	78 kDa glucose-regulated protein	CRP78	P11021	-8.40
3NCL	Suppressor of tumorigenicity 14 protein	ST14	Q9Y5Y6	-8.40

3O0G	Cell division protein kinase 5	CDK5	Q00535	-8.40
5M6U	Phosphatidylinositol 4,5-bisphosphate 3-kinase catalytic subunit delta isoform	PIK3CD	O00329	-8.40
6GU2	Cyclin-dependent kinase 1	CDK1	P06493	-8.40
1HY7	Stromelysin 1	MMP3	P08254	-8.30
2W7X	Serine/threonine-protein kinase Chk2	CHK2	O96017	-8.30
3I33	Heat shock-related 70 kDa protein 2	HSPA2	P54652	-8.30
3KMW	Integrin-linked protein kinase	ILK	Q13418	-8.30
5UJB	Serum albumin	ALB	P02768	-8.30
1AU8	Cathepsin G	CTSG	P08311	-8.20
1HOV	Matrix metalloproteinase 2	MMP2	P08253	-8.20
1LYW	Cathepsin D	CTSD	P07339	-8.20
2F2C	Cell division protein kinase 6	CDK6	Q00534	-8.20
3DKO	Ephrin type-A receptor 7	EPHA7	Q15375	-8.20
3KMM	Tyrosine-protein kinase LCK	LCK	P06239	-8.20
3LXP	Non-receptor tyrosine-protein kinase TYK2	TYK2	P29597	-8.20
4B9D	Serine/Threonine-protein kinase NEK1	NEK1	Q96PY6	-8.20
2IZR	Casein kinase I isoform gamma-3	CSNK1G3	Q9Y6M4	-8.10
2JC6	Calcium/calmodulin-dependent protein kinase type 1D	CAMK1D	Q8IU85	-8.10
3BQR	Death-associated protein kinase 3	DAPK3	O43293	-8.10
3BZ3	Focal adhesion kinase 1	FAK1	Q05397	-8.10
3DHE	Estrogen 17 beta-hydroxysteroid dehydrogenase	HSD17B1	P14061	-8.10
3ENE	Phosphatidylinositol-4,5-bisphosphate 3-kinase catalytic subunit gamma isoform	PIK3CG	P48736	-8.10
3FE1	Heat shock 70 kDa protein 6	HSPA6	P17066	-8.10
4BTJ	Tau-tubulin kinase 1	TTBK1	Q5TCY1	-8.10
4CRS	Serine/threonine-protein kinase N2	PKN2	Q16513	-8.10
4Z07	cGMP-dependent protein kinase 1	PRKG1	Q13976	-8.10
5BVW	Epithelial discoidin domain-containing receptor 1	DDR1	Q08345	-8.10
6BFN	Serine/threonine-protein kinase PLK1	PLK1	P53350	-8.10
1SU3	Interstitial collagenase	MMP1	P03956	-8.00
3EH9	Death-associated protein kinase 1	DAPK1	P53355	-8.00
3F5P	Insulin-like growth factor 1 receptor	IGF1R	P08069	-8.00
3IUC	Heat shock 70kDa protein 5	HSPA5	P11021	-8.00
4KS7	Serine/threonine-protein kinase PAK 6	PAK6	Q9NQU5	-8.00
830C	Collagenase 3	MMP13	P45452	-8.00
1GMY	Cathepsin B	CTSB	P07858	-7.90
1WBP	Serine/threonine protein kinase SPRK1	SRPK	Q96SB4	-7.90
2J51	Ste20-like serine/threonine protein kinase	SLK	Q9H2G2	-7.90
2VIP	Urokinase-type plasminogen activator B chain	PLAU	P00749	-7.90
3COK	Serine/threonine-protein kinase PLK4	PLK4	O00444	-7.90

3DZQ	EPH receptor A3	EphA3	P29320	-7.90
3FZS	Protein tyrosine kinase 2 beta	PTK2B	Q14289	-7.90
3LM0	Serine/threonine-protein kinase 17B	STK17B	O94768	-7.90
5QBU	Cathepsin S	CTSS	P25774	-7.90
2ISI	DNA-(apurinic or apyrimidinic site) lyase	APEX1	P27695	-7.80
2O21	Apoptosis regulator Bcl-2	BCL2	P10415	-7.80
2XBA	ALK tyrosine kinase receptor	ALK	Q9UM73	-7.80
2ZOQ	Mitogen-activated protein kinase 3	MAPK3	P27361	-7.80
3ZBF	Proto-oncogene tyrosine-protein kinase ROS	ROS1	P08922	-7.80
1XW6	Glutathione S-transferase Mu1	GSTM1	P09488	-7.70
2OO8	Angiopoietin-1 receptor	TEK	Q02763	-7.70
3E7O	Mitogen-activated protein kinase 9	JNK2	P45984	-7.70
3EWH	Vascular endothelial growth factor receptor 2	VEGFR2	P35968	-7.70
3G0E	Mast/stem cell growth factor receptor	KIT	P10721	-7.70
3HMO	Dual specificity protein kinase TTK	TTK	P33981	-7.70
4ACS	Glutathione S-transferase A2	GSTA2	P09210	-7.70
4UXQ	FGF receptor 4	FGFR4	P22455	-7.70
6G0W	Poly [ADP-ribose] polymerase 14	PARP14	Q460N5	-7.70
2X4Z	Serine/threonine-protein kinase PAK 4	PAK4	O96013	-7.60
3DDU	Prolyl endopeptidase	PREP	P48147	-7.60
3Q4Z	Serine/threonine-protein kinase PAK 1	PAK1	Q13153	-7.60
4C8B	Receptor-interacting serine/threonine-protein kinase 2	RIPK2	O43353	-7.60
1YFK	Phosphoglycerate mutase 1	PGAM1	P18669	-7.50
2BR9	14-3-3 protein epsilon	YWHAE	P62258	-7.50
3KA0	MAP kinase-activated protein kinase 2	MK2	P49137	-7.50
5GRN	Histone deacetylase 1	HDAC1	Q13547	-7.50
6EZP	Cathepsin H	CTSH	P09668	-7.50
2HV7	Serine/threonine-protein phosphatase 2A activator	PTPA	Q15257	-7.40
5XG8	Galectin13	LGALS13	Q9UHV8	-7.40
1BXL	Bcl-2-like protein 1	BCLXL	Q07817	-7.30
3GUR	Glutathione S-transferase Mu 2	GSTM2	P28161	-7.30
1YVL	Signal transducer and activator of transcription 1	STAT1	P42224	-7.20
3BKY	B-lymphocyte antigen CD20	CD20	P11836	-7.20
3DTC	Mitogen-activated protein kinase kinase kinase 9	MAP3K9	P80192	-7.20
3HWN	Cathepsin L	CTSL	P07711	-7.20
3O5R	Peptidyl-prolyl cis-trans isomerase FKBP5	FKBP5	Q13451	-7.20
4C57	Cyclin-G-associated kinase	GAK	O14976	-7.20
4H82	Matrix metalloproteinase-9	MMP9	P14780	-7.20
4RT7	Receptor-type tyrosine-protein kinase FLT3	FLT3	P36888	-7.20
1K4T	DNA topoisomerase I	TOPI	P11387	-7.10

1LD8	Protein farnesyltransferase alpha subunit	FNTA	P49354	-7.10
1TDI	Glutathione S-transferase A3	GSTA3	Q16772	-7.10
2D06	Sulfotransferase 1A1	SULT1A1	P50225	-7.10
2X4F	Myosin light chain kinase family member 4	MYLK4	Q86YV6	-7.10
3EWT	Calmodulin	CALM2	P0DP24	-7.10
3TXO	Protein kinase C eta	PRKCH	P24723	-7.10
4ZSG	Mitogen-activated protein kinase 7	MAPK7	Q13164	-7.10
1M6D	Cathepsin F	CTSF	Q9UBX1	-7.00
2EJR	Lysine-specific histone demethylase 1	LSD1	O60341	-7.00
2OY2	Neutrophil collagenase	MMP8	P22894	-7.00
2R6N	Cathepsin K	CTSK	P43235	-7.00
3IK7	Glutathione S-transferase A4	GSTA4	O15217	-7.00
3OOB	Peptidyl-prolyl cis-trans isomerase NIMA-interacting 1	Pin1	Q13526	-7.00
4LAY	Peptidyl-prolyl cis-trans isomerase FKBP4	FKBP4	Q02790	-7.00
1YWT	14-3-3 protein sigma	SFN	P31947	-6.90
4W9W	BMP-2-inducible protein kinase	BMP2K	Q9NSY1	-6.90
5J3S	Tyrosyl-DNA phosphodiesterase 2	TDP2	O95551	-6.90
2BTP	14-3-3 PROTEIN TAU	YWHAQ	P27348	-6.80
2HOG	Serine/threonine-protein kinase Chk1	Chek1	O14757	-6.80
3A1B	DNA (cytosine-5)-methyltransferase 3A	DNMT3A	P68431	-6.80
3VKL	Galectin8	LGALS8	O00214	-6.80
5FCW	Platelet-derived growth factor receptor alpha	PDGFRA	P16234	-6.80
2PCU	Carboxypeptidase A4	CPA4	Q9UI42	-6.70
5IWG	Histone deacetylase 2	HDAC2	Q92769	-6.70
5O7P	Receptor tyrosine-protein kinase erbB-3	ERBB3	P21860	-6.70
1D5R	Phosphoinstide phosphatase PTEN	PTEN	P60484	-6.60
1DTD	Carboxypeptidase A2	CPA2	P48052	-6.60
1KJL	Galectin3	LGALS3	P17931	-6.60
1S78	Receptor protein-tyrosine kinase erbB-2	ErbB2	P04626	-6.60
2F1O	NAD(P)H dehydrogenase [quinone] 1	NQO1	P15559	-6.60
3FAP	FK506-binding protein	FKBP1A	P62942	-6.60
3LBZ	B-cell lymphoma 6 protein	BCL6	P41182	-6.60
3WV6	Galectin9	LGALS9	O00182	-6.60
5ICN	Peroxiredoxin-2	PRDX2	P32119	-6.60
6FYZ	Histone deacetylase 4	HDAC4	P56524	-6.60
6I6Z	Carboxypeptidase A1	CPA1	P15085	-6.60
1DI9	Mitogen-activated protein kinase 14	MAPK14	Q16539	-6.50
1NMK	Peptidyl-prolyl cis-trans isomerase A	PPIA	P62937	-6.50
1UOU	Thymidine phosphorylase	TYMP	P19971	-6.50
2X7F	TRAF2 and NCK-interacting protein kinase	TNIK	Q9UKE5	-6.50
4DRI	Serine/threonine-protein kinase mTOR	MTOR	P42345	-6.50
4R7H	Macrophage colony-stimulating factor 1 receptor	CSF1R	P07333	-6.50

5WT9	Programmed cell death protein 1	PD1	Q15116	-6.50
1C9H	Peptidyl-prolyl cis-trans isomerase FKBP1B	FKBP1B	P68106	-6.40
1EF7	Cathepsin X	CTSX	Q9UBR2	-6.40
3EQS	E3 ubiquitin-protein ligase MDM2	MDM2	Q00987	-6.40
4KNM	Carbonic anhydrase 13	CA13	Q8N1Q1	-6.40
4YLZ	Galectin4	LGALS4	P56470	-6.40
1QJA	14-3-3 protein ZETA	ZETA	P63104	-6.30
1SHL	Caspase7	CASP7	P55210	-6.30
3GAL	Galectin7	LGALS7	P47929	-6.30
3HMI	Tyrosine-protein kinase ABL2	ABL2	P42684	-6.30
4GU0	Lysine-specific histone demethylase 1B	LSD2	Q8NB78	-6.30
5LCZ	Glutathione S-transferase A1	GSTA1	P08263	-6.30
1SVC	Protein nuclear factir kappa-B	NFKB1	P19838	-6.20
1ZRZ	Protein kinase C iota	PRKCI	P41743	-6.20
2RAW	Baculoviral IAP repeat-containing protein 5	BIRC5	O15392	-6.20
2WFY	Hepatocyte growth factor receptor	MET	P08581	-6.20
3MJG	platelet-derived growth factor receptor beta	PDGFRB	P09619	-6.20
5DG1	Galectin2	LGALS2	P05162	-6.20
5VL3	B-cell receptor CD22	CD22	P20273	-6.20
6AL5	Casein kinase II subunit alpha	CSNK2A1	P68400	-6.20
1W6M	Galectin1	LGALS1	P09382	-6.10
2VM5	Baculoviral IAP repeat-containing protein 1	BIRC1	Q13075	-6.10
2VM6	BCL-2-RELATED PROTEIN A1	BCL2A1	Q16548	-6.10
3NAX	3-phosphoinositide-dependent protein kinase 1	PDPK1	O15530	-6.10
4RS1	Granulocyte-macrophage colony-stimulating factor receptor subunit alpha	CSF2RA	P15509	-6.10
5XRG	Galectin10	LGALS10	Q05315	-6.10
6CZK	Nitric oxide synthase, brain	NNOS	P29475	-6.10
1RFF	Tyrosyl-DNA phosphodiesterase 1	TDP1	Q9NUW8	-6.00
2CBZ	Multidrug resistance-associated protein 1	MRP1	P33527	-6.00
3H6S	Cathepsin V	CTSV	O60911	-6.00
1PD8	Dihydrofolate reductase	DHFR	P00374	-5.90
3D9U	Baculoviral IAP repeat-containing protein 2	BIRC2	Q13490	-5.90
3EB5	Baculoviral IAP repeat-containing protein 3	BIRC3	Q13489	-5.90
3KR7	Tankyrase-2	TNKS2	Q9H2K2	-5.90
2O72	Epithelial-cadherin	CDH1	P12830	-5.80
2UZK	Forkhead box protein O3A	FOXO3	O43524	-5.80
2X18	RAC-gamma serine/threonine-protein kinase	AKT3	Q9Y243	-5.80
4A69	Histone deacetylase 3	HDAC3	O15379	-5.80
4XCS	Peroxiredoxin-1	PRDX1	Q06830	-5.80
3EDQ	Caspase3	CASP3	P42574	-5.70
1QTN	Caspase8	CASP8	Q14790	-5.60
2FQQ	Caspase-1	CASP1	P29466	-5.60

2W3O	Bifunctional polynucleotide phosphatase/kinase	PNKP	Q96T60	-5.60
3BIK	Programmed cell death 1 ligand 1	PDL1	Q9NZQ7	-5.60
5J06	Myeloid cell surface antigen CD33	CD33	P20138	-5.60
5T89	Vascular endothelial growth factor receptor 1	VEGFR1	P17948	-5.60
2JVN	Poly [ADP-ribose] polymerase 1	PARP1	P09874	-5.50
2VL2	Peroxiredoxin-5	PRDX5	P30044	-5.40
3EQY	Protein Mdm4	MDM4	O15151	-5.40
2K05	Stromal cell-derived factor 1	CXCL12	P48061	-5.30
2O2U	Mitogen-activated protein kinase 10	JNK3	P53779	-5.20
1HKN	Heparin-binding growth factor 1	FGF1	P05230	-5.10
1Z57	Dual specificity protein kinase CLK1	CLK1	P49759	-5.00
3D7V	Induced myeloid leukemia cell differentiation protein Mcl-1	MCL1	Q07820	-5.00
3GRW	FGF receptor 3	FGFR3	P22607	-5.00
3REY	Adenosine receptor A2a	ADORA2A	P29274	-5.00
3L2C	Forkhead box protein O4	FOXO4	P98177	-4.90
4OEE	Fibroblast growth factor 2	FGF2	P09038	-4.80
1EV2	FGF receptor 2	FGFR2	P21802	-4.60
1ZY3	Apoptosis regulator Bcl-W	BCLW	Q92843	-4.10
3D67	Carboxypeptidase B2	CPB2	Q96IY4	-2.30
3NYN	G protein-coupled receptor kinase 6	GRK6	P43250	-0.20
3F1Q	Dihydroorotate dehydrogenase	DHODH	Q02127	0.00

Table S3. The results of KEGG pathway enrichment

Pathways	ID	Input	Background	P-Value
Apoptosis	hsa04210	10	208	5.32E-13
Neurotrophin signaling pathway	hsa04722	9	200	1.55E-11
Pathways in cancer	hsa05200	9	607	1.94E-07
FoxO signaling pathway	hsa04068	8	208	7.8E-10
Hepatitis B	hsa05161	8	214	9.68E-10
Tuberculosis	hsa05152	8	268	5.36E-09
Epstein-Barr virus infection	hsa05169	8	312	1.7E-08
MAPK signaling pathway	hsa04010	8	375	6.79E-08
Chagas disease	hsa05142	7	170	6.3E-09
HTLV-I infection	hsa05166	7	399	1.77E-06
PI3K-Akt signaling pathway	hsa04151	7	512	8.89E-06
Fc epsilon RI signaling pathway	hsa04664	6	105	1.3E-08
Small cell lung cancer	hsa05222	6	136	5.67E-08
ErbB signaling pathway	hsa04012	6	141	6.96E-08
Progesterone-mediated oocyte maturation	hsa04914	6	144	7.85E-08
TNF signaling pathway	hsa04668	6	154	1.15E-07
Toll-like receptor signaling pathway	hsa04620	6	155	1.2E-07
Toxoplasmosis	hsa05145	6	195	4.42E-07
Measles	hsa05162	6	198	4.82E-07
Influenza A	hsa05164	6	259	2.21E-06
Ras signaling pathway	hsa04014	6	336	9.53E-06
Acute myeloid leukemia	hsa05221	5	88	2.39E-07
Pancreatic cancer	hsa05212	5	111	7.19E-07
B cell receptor signaling pathway	hsa04662	5	112	7.5E-07
Adipocytokine signaling pathway	hsa04920	5	113	7.83E-07
Chronic myeloid leukemia	hsa05220	5	120	1.04E-06
Platinum drug resistance	hsa01524	5	125	1.26E-06
Prostate cancer	hsa05215	5	139	2.09E-06
Insulin resistance	hsa04931	5	157	3.73E-06
AGE-RAGE signaling pathway in diabetic complications	hsa04933	5	163	4.46E-06
T cell receptor signaling pathway	hsa04660	5	166	4.86E-06
Sphingolipid signaling pathway	hsa04071	5	184	7.91E-06
Osteoclast differentiation	hsa04380	5	184	7.91E-06
Cell cycle	hsa04110	5	192	9.67E-06
Hepatitis C	hsa05160	5	193	9.91E-06
Signaling pathways regulating pluripotency of stem cells	hsa04550	5	205	1.32E-05
Insulin signaling pathway	hsa04910	5	214	1.61E-05
Non-alcoholic fatty liver disease (NAFLD)	hsa04932	5	226	2.08E-05
Chemokine signaling pathway	hsa04062	5	285	6.16E-05
Colorectal cancer	hsa05210	4	108	2.14E-05
Central carbon metabolism in cancer	hsa05230	4	111	2.38E-05
Prolactin signaling pathway	hsa04917	4	118	3E-05

NF-kappa B signaling pathway	hsa04064	4	137	5.28E-05
Choline metabolism in cancer	hsa05231	4	149	7.25E-05
Endocrine resistance	hsa01522	4	159	9.27E-05
Tight junction	hsa04530	4	197	0.000207
Jak-STAT signaling pathway	hsa04630	4	214	0.000283
mTOR signaling pathway	hsa04150	4	217	0.000298
Phospholipase D signaling pathway	hsa04072	4	221	0.000319
MicroRNAs in cancer	hsa05206	4	235	0.0004
Viral carcinogenesis	hsa05203	4	328	0.001363
Amyotrophic lateral sclerosis (ALS)	hsa05014	3	83	0.00027
Endometrial cancer	hsa05213	3	88	0.000319
Non-small cell lung cancer	hsa05223	3	92	0.000362
VEGF signaling pathway	hsa04370	3	98	0.000433
p53 signaling pathway	hsa04115	3	99	0.000446
Epithelial cell signaling in Helicobacter pylori infection	hsa05120	3	103	0.000499
Shigellosis	hsa05131	3	104	0.000513
Glioma	hsa05214	3	107	0.000556
Melanoma	hsa05218	3	107	0.000556
Longevity regulating pathway - multiple species	hsa04213	3	113	0.000649
Renal cell carcinoma	hsa05211	3	116	0.000699
Pertussis	hsa05133	3	117	0.000716
EGFR tyrosine kinase inhibitor resistance	hsa01521	3	121	0.000788
Fc gamma R-mediated phagocytosis	hsa04666	3	144	0.001288
Longevity regulating pathway	hsa04211	3	150	0.001445
Inflammatory mediator regulation of TRP channels	hsa04750	3	152	0.001499
GnRH signaling pathway	hsa04912	3	152	0.001499
HIF-1 signaling pathway	hsa04066	3	170	0.002052
Estrogen signaling pathway	hsa04915	3	170	0.002052
Thyroid hormone signaling pathway	hsa04919	3	178	0.002333
Cholinergic synapse	hsa04725	3	180	0.002406
Platelet activation	hsa04611	3	182	0.002482
Oocyte meiosis	hsa04114	3	185	0.002597
Dopaminergic synapse	hsa04728	3	207	0.003547
Herpes simplex infection	hsa05168	3	273	0.007564
Rap1 signaling pathway	hsa04015	3	315	0.011111
Proteoglycans in cancer	hsa05205	3	316	0.011205
Focal adhesion	hsa04510	3	321	0.011684
cAMP signaling pathway	hsa04024	3	323	0.011878
Arginine biosynthesis	hsa00220	2	35	0.001338
Apoptosis - multiple species	hsa04215	2	46	0.002244
Base excision repair	hsa03410	2	54	0.003041
Thyroid cancer	hsa05216	2	55	0.003148
Carbohydrate digestion and absorption	hsa04973	2	68	0.004708
Arginine and proline metabolism	hsa00330	2	75	0.005667

NOD-like receptor signaling pathway	hsa04621	2	80	0.006403
Type II diabetes mellitus	hsa04930	2	82	0.006709
RIG-I-like receptor signaling pathway	hsa04622	2	87	0.007502
Regulation of lipolysis in adipocytes	hsa04923	2	89	0.00783
Long-term depression	hsa04730	2	104	0.010496
Leishmaniasis	hsa05140	2	111	0.011859
Gap junction	hsa04540	2	131	0.016153
Salmonella infection	hsa05132	2	132	0.016383
Glucagon signaling pathway	hsa04922	2	163	0.024183
AMPK signaling pathway	hsa04152	2	189	0.031675
Natural killer cell mediated cytotoxicity	hsa04650	2	202	0.035723
Adrenergic signaling in cardiomyocytes	hsa04261	2	230	0.045073

Table S4. The center box of anti-tumor targets for AutoDock Vina.

PDB_ID	Gene_Name	Uniprot_ID	Center_X	Center_Y	Center_Z
1AD5	HCK	P08631	26.00	47.00	100.00
1AGW	FGFR1	P11362	14.00	3.00	20.30
1AU8	CTSG	P08311	11.00	43.00	4.30
1BXL	BCLXL	Q07817	7.27	-11.81	0.05
1C9H	FKBP1B	P68106	-22.00	-15.00	-13.00
1D5R	PTEN	P60484	37.27	88.19	25.05
1DI9	MAPK14	Q16539	45.00	33.00	27.00
1DTD	CPA2	P48052	45.00	45.00	1.00
1EF7	CTSX	Q9UBR2	51.00	14.00	125.00
1EV2	FGFR2	P21802	82.04	7.53	-13.77
1GMY	CTSB	P07858	34.00	36.00	39.30
1HKN	FGF1	P05230	16.00	20.00	81.30
1HOV	MMP2	P08253	4.00	19.00	23.00
1HVY	TYMS	P04818	0.68	10.10	19.06
1HY7	MMP3	P08254	1.00	52.00	52.30
1K4T	TOPI	P11387	21.00	-1.00	28.30
1KJL	LGALS3	P17931	-3.48	-11.35	0.68
1LD8	FNTA	P49354	17.78	139.82	-1.70
1LYW	CTSD	P07339	149.00	10.00	83.00
1M6D	CTSF	Q9UBX1	3.00	9.00	0.00
1M6I	AIF	O95831	3.56	53.02	22.54
1NMK	PPIA	P62937	34.00	3.00	43.00
1PD8	DHFR	P00374	29.78	10.82	6.30
1QJA	ZETA	P63104	22.78	-6.18	36.30
1QTN	CASP8	Q14790	-6.22	30.82	19.30
1RFF	TDP1	Q9NUW8	3.78	51.82	-14.70
1S78	ErbB2	P04626	59.78	82.82	227.30
1SHL	CASP7	P55210	51.28	17.55	1.95
1SU3	MMP1	P03956	16.00	-103.00	12.00
1SVC	NFKB1	P19838	26.29	28.89	36.57
1TDI	GSTA3	Q16772	13.00	13.00	43.00
1U59	ZAP70	P43403	7.00	4.00	54.00
1UA2	CDK7	P50613	39.28	-7.45	21.95
1UOU	TYMP	P19971	-11.00	1.00	28.00
1W6M	LGALS1	P09382	5.78	59.82	27.30
1WBP	SRPK	Q96SB4	26.27	20.58	28.21
1X8B	WEE1	P30291	5.27	54.58	26.21
1XBC	SYK	P43405	3.00	-1.00	16.00
1XW6	GSTM1	P09488	-10.00	9.00	75.00
1YFK	PGAM1	P18669	3.27	-4.42	51.21

1YVJ	JAK3	P52333	8.27	-10.42	-1.79
1YVL	STAT1	P42224	-28.00	-19.00	147.00
1YW9	METAP2	P50579	13.27	26.58	15.21
1YWT	SFN	P31947	26.00	40.00	64.00
1Z57	CLK1	P49759	13.27	26.58	15.21
1Z6T	APAF1	O14727	-0.73	44.58	69.21
1ZRZ	PRKCI	P41743	-10.00	19.00	18.00
1ZXM	TOPII	P11388	34.27	-0.42	36.21
1ZY3	BCLW	Q92843	8.27	2.58	26.21
2A2R	GSTP1	P09211	8.27	2.58	26.21
2BR9	YWHAE	P62258	-18.73	3.58	19.21
2BTP	YWHAQ	P27348	28.27	-26.42	30.21
2CBZ	MRP1	P33527	-15.73	47.58	-1.79
2CLQ	MAP3K5	Q99683	-1.00	9.00	-26.00
2D06	SULT1A1	P50225	136.00	-63.00	1.00
2DQ7	FYN	P06241	-13.00	17.00	-11.00
2EJR	LSD1	O60341	21.27	43.58	33.21
2F1O	NQO1	P15559	16.27	17.58	-0.79
2F2C	CDK6	Q00534	30.27	18.58	61.21
2FQQ	CASP1	P29466	56.27	25.58	40.21
2HOG	Chek1	O14757	18.00	0.00	9.00
2HV7	PTPA	Q15257	11.00	-33.00	17.00
2I0E	PRKCB	P05771	42.00	56.00	32.00
2ISI	APEX1	P27695	31.27	6.58	41.21
2IVT	RET	P07949	62.00	9.00	9.00
2IW9	CDK2	P24941	6.27	40.58	50.21
2IZR	CSNK1G3	Q9Y6M4	9.00	30.00	25.00
2J51	SLK	Q9H2G2	-28.00	49.00	-6.00
2J6M	EGFR	P00533	-52.97	3.58	-20.63
2J8Z	PIG3	Q53FA7	27.00	31.00	-23.00
2JAM	CAMK1G	Q96NX5	15.00	5.00	12.00
2JC6	CAMK1D	Q8IU85	-10.00	-79.00	-37.00
2JDR	AKT2	P31751	23.00	7.00	41.00
2JED	PRKCQ	Q04759	58.00	18.00	-5.00
2JVN	PARP1	P09874	0.03	9.58	11.37
2K05	CXCL12	P48061	4.03	8.58	4.37
2NRU	IRAK4	Q9NWZ3	28.00	8.00	-5.00
2O21	BCL2	P10415	4.03	8.58	4.37
2O2U	JNK3	P53779	18.00	8.00	32.00
2O72	CDH1	P12830	37.03	-16.42	42.37
2OJG	MAPK1	P28482	-13.97	10.58	42.37
2OO8	TEK	Q02763	47.03	-2.42	74.37
2OY2	MMP8	P22894	-0.97	3.58	5.37
2PCU	CPA4	Q9UI42	1.00	40.00	16.00

2QRV	DNMT3L	Q9UJW3	107.17	50.77	3.54
2R4B	ERBB4	Q15303	-17.00	16.00	-2.00
2R6N	CTSK	P43235	31.17	4.77	7.54
2RAW	BIRC5	O15392	8.35	10.54	10.52
2UZK	FOXO3	O43524	7.37	-7.94	-4.23
2V7A	ABL1	P00519	-42.00	-55.00	-10.00
2VD5	DMPK	Q09013	66.00	-43.00	14.00
2VIP	PLAU	P00749	23.17	8.77	29.54
2VL2	PRDX5	P30044	8.00	7.00	41.00
2VM5	BIRC1	Q13075	-7.00	4.00	-4.00
2VM6	BCL2A1	Q16548	11.17	20.77	62.54
2VX1	EphB4	P54760	12.00	9.00	11.00
2W3O	PNKP	Q96T60	29.00	-4.00	-9.00
2W7X	CHK2	O96017	49.04	-13.87	-7.36
2WFY	MET	P08581	41.04	24.13	3.64
2WGJ	HSP90AA1	P07900	23.04	81.13	3.64
2WI6	TGFBR1	P36897	34.04	8.13	26.64
2WOU	NEK7	Q8TDX7	3.00	7.00	5.00
2WU6	CLK3	P49761	34.04	56.13	15.64
2X18	AKT3	Q9Y243	25.00	95.00	20.00
2X4F	MYLK4	Q86YV6	94.00	52.00	28.00
2X4Z	PAK4	O96013	20.00	20.00	56.00
2X7F	TNIK	Q9UKE5	31.00	-4.00	58.00
2X7G	SRPK2	P78362	-26.00	43.00	-17.00
2XBA	ALK	Q9UM73	4.00	15.00	6.00
2XIK	STK25	O00506	-2.03	-9.77	-22.31
2XKF	NEK2	P51955	-28.00	21.00	-20.00
2XXZ	KDM6B	O15054	41.00	44.00	8.00
2Z7Q	RPS6KA1	Q15418	2.00	-7.00	23.00
2ZOQ	MAPK3	P27361	30.86	5.09	15.55
2ZV2	CAMKK2	Q96RR4	1.00	-5.00	-28.00
3A1B	DNMT3A	P68431	30.04	-0.87	15.64
3A60	RPS6KB1	P23443	-6.00	4.00	38.00
3A7H	STK24	Q9Y6E0	4.00	28.00	46.00
3ALN	MAP2K4	P45985	17.00	-1.00	-21.00
3AMB	PKACA	P17612	-28.03	-10.23	-4.31
3BHH	CamKIIB	Q13554	17.00	66.00	32.00
3BIK	PDL1	Q9NZQ7	10.04	-9.87	-36.36
3BIY	EP300	Q09472	-20.00	18.00	2.00
3BKY	CD20	P11836	8.04	3.13	-23.36
3BLQ	CDK9	P50750	48.04	-21.87	-10.36
3BQR	DAPK3	O43293	3.00	44.00	34.00
3BZ3	FAK1	Q05397	12.04	2.13	6.64
3C0G	CASK	O14936	5.00	8.00	-6.00

3C4C	BRAF	P15056	-0.30	-4.53	-16.78
3CBL	FES	P07332	24.00	21.00	23.00
3COK	PLK4	O00444	35.00	61.00	34.00
3D67	CPB2	Q96IY4	39.00	38.00	42.00
3D7V	MCL1	Q07820	3.70	-18.53	17.22
3D9U	BIRC2	Q13490	-1.30	21.47	10.22
3DDU	PREP	P48147	-11.30	14.47	31.22
3DHE	HSD17B1	P14061	13.97	12.77	-10.69
3DKO	EPHA7	Q15375	-17.00	12.00	-12.00
3DTC	MAP3K9	P80192	19.97	38.77	26.31
3DV3	MEK1	Q02750	45.70	-11.53	1.22
3DZQ	EphA3	P29320	-6.00	14.00	55.00
3E64	JAK2	O60674	34.70	39.47	35.22
3E7O	JNK2	P45984	-33.97	-31.77	27.31
3EB5	BIRC3	Q13489	2.70	27.47	30.22
3EDQ	CASP3	P42574	-2.30	-3.53	10.22
3EH9	DAPK1	P53355	-23.00	0.00	-12.00
3ELJ	JNK1	P45983	25.97	8.23	30.31
3ENE	PIK3CG	P48736	41.98	8.08	29.93
3EQR	ACK1	Q07912	26.97	8.23	23.31
3EQS	MDM2	Q00987	-8.02	0.08	-0.93
3EQY	MDM4	O15151	-7.02	29.08	-0.93
3EWH	VEGFR2	P35968	15.98	-2.92	12.07
3EWT	CALM2	P0DP24	23.98	21.08	37.07
3EYG	JAK1	P23458	10.98	21.08	-15.07
3F1Q	DHODH	Q02127	0.00	-43.00	6.00
3F5P	IGF1R	P08069	82.98	51.08	149.93
3F7Z	GSK3B	P49841	-0.02	12.08	14.93
3FAP	FKBP1A	P62942	-7.00	25.00	36.00
3FE1	HSPA6	P17066	4.00	-5.00	23.00
3FZH	HSPA8	P11142	19.00	-5.00	1.00
3FZS	PTK2B	Q14289	-2.02	-2.92	9.93
3G0E	KIT	P10721	35.00	-4.00	-77.00
3GAL	LGALS7	P47929	9.00	2.00	30.00
3GDQ	HSPA1L	P34931	12.00	11.00	10.00
3GFT	KRAS	P01116	64.98	114.31	2.75
3GRW	FGFR3	P22607	41.76	22.68	-41.69
3GUR	GSTM2	P28161	11.00	-6.00	12.00
3H6S	CTSV	O60911	36.00	54.00	25.00
3HIG	AOC1	P19801	-26.24	-11.32	74.69
3HMI	ABL2	P42684	-30.24	7.32	-17.69
3HMO	TTK	P33981	-35.00	-13.00	-16.00
3HNA	EHMT1	Q9H9B1	40.24	7.32	7.69
3HWN	CTSL	P07711	26.76	-26.32	-12.31

3I33	HSPA2	P54652	-15.00	-5.00	9.00
3IK7	GSTA4	O15217	9.00	14.00	19.00
3IUC	HSPA5	P11021	2.00	-7.00	5.00
3IW4	PRKCA	P17252	4.00	31.00	52.00
3JXU	HSPA1A	P0DMV8	-6.00	-14.00	19.00
3JYA	PIM1	P11309	3.76	82.68	0.69
3KA0	MK2	P49137	236.00	84.00	182.00
3KMM	LCK	P06239	28.00	37.00	84.00
3KMW	ILK	Q13418	-2.00	-3.00	11.00
3KN5	RPS6KA5	O75582	24.00	38.00	74.00
3KR7	TNKS2	Q9H2K2	24.00	4.00	13.00
3KRW	GRK2	P25098	34.00	-5.00	53.00
3L2C	FOXO4	P98177	5.37	8.06	-11.23
3LBZ	BCL6	P41182	5.00	-23.00	15.00
3LDP	CRP78	P11021	17.00	-12.00	8.00
3LM0	STK17B	O94768	19.00	36.00	1.00
3LXP	TYK2	P29597	-7.00	28.00	-30.00
3MDY	FKBP1A	P62942	43.00	13.00	59.00
3MJG	PDGFRB	P09619	35.00	-68.00	-3.00
3MTF	ACVR1	Q04771	-17.00	-10.00	40.00
3MVH	AKT1	P31749	19.76	-4.32	30.69
3NAX	PDPK1	O15530	10.00	-5.00	4.00
3NCL	ST14	Q9Y5Y6	21.00	26.00	29.00
3NR9	CLK2	P49760	79.00	-1.00	-7.00
3NYN	GRK6	P43250	-47.00	58.00	-46.00
3O0G	CDK5	Q00535	-6.00	62.00	57.00
3O5R	FKBP5	Q13451	52.00	15.00	20.00
3OOB	Pin1	Q13526	31.00	28.00	20.00
3OP5	VRK1	Q99986	31.00	2.00	18.00
3P23	ERN1	O75460	24.00	-38.00	46.00
3PJ1	BTK	Q06187	-25.00	6.00	-19.00
3PLS	MST1R	Q04912	31.00	23.00	6.00
3Q4Z	PAK1	Q13153	14.00	-19.00	14.00
3QFV	CDC42BPB	Q9Y5S2	-7.00	-71.00	20.00
3REY	ADORA2A	P29274	47.00	23.00	36.00
3T9T	ITK	Q08881	-7.00	4.00	11.00
3TXO	PRKCH	P24723	22.00	10.00	14.00
3UYT	CSNK1D	P48730	-7.00	-6.00	-11.00
3V8S	ROCK1	Q13464	-42.00	6.00	29.00
3VKL	LGALS8	O00214	-10.00	1.00	-14.00
3WV6	LGALS9	O00182	46.00	-9.00	30.00
3ZBF	ROS1	P08922	47.00	18.00	3.00
3ZNR	HDAC7	Q8WUI4	-8.00	7.00	-16.00
4A69	HDAC3	O15379	19.00	48.00	27.00

4ACS	GSTA2	P09210	14.00	12.00	1.00
4AF3	AURKB	Q96GD4	20.00	-23.00	-13.00
4AOJ	NTRK1	P04629	96.00	53.00	25.00
4AT3	NTRK2	Q16620	9.00	-25.00	-12.00
4B6L	PLK3	Q9H4B4	56.00	-4.00	-11.00
4B9D	NEK1	Q96PY6	6.00	13.00	-27.00
4BC6	STK10	O94804	-5.00	-19.00	22.00
4BTJ	TTBK1	Q5TCY1	-5.00	10.00	-36.00
4C57	GAK	O14976	-28.00	10.00	41.00
4C8B	RIPK2	O43353	8.00	-8.00	-4.00
4CRS	PKN2	Q16513	-9.00	33.00	191.00
4DRI	MTOR	P42345	38.00	49.00	37.00
4G31	EIF2AK3	Q9NZJ5	-49.00	9.00	5.00
4GU0	LSD2	Q8NB78	-64.00	-28.00	-11.00
4GV0	PARP3	Q9Y6F1	20.00	-2.00	12.00
4H82	MMP9	P14780	2.00	-3.00	30.00
4I5M	PLK2	Q9NYY3	11.00	9.00	9.00
4IFC	PRPF4B	Q13523	-3.00	4.00	15.00
4IM0	TBK1	Q9UHD2	111.00	15.00	-25.00
4K4E	TNKS1	O95271	-9.00	38.00	28.00
4KIK	IKBKB	O14920	47.00	30.00	-58.00
4KNM	CA13	Q8N1Q1	3.00	52.00	13.00
4KS7	PAK6	Q9NQU5	32.00	37.00	27.00
4LAY	FKBP4	Q02790	21.00	-17.00	16.00
4MQ1	DYRK1A	Q13627	40.00	21.00	-40.00
4NOS	NOS2	P35228	5.00	91.00	15.00
4OEE	FGF2	P09038	-10.00	-8.00	-3.00
4OEL	CTSC	P53634	34.00	20.00	13.00
4OTP	RIOK1	Q9BRS2	28.00	44.00	31.00
4R7H	CSF1R	P07333	-21.00	5.00	16.00
4R8Q	BUB1	O43683	-31.00	18.00	-26.00
4RS1	CSF2RA	P15509	5.00	-23.00	-15.00
4RT7	FLT3	P36888	-42.00	13.00	-17.00
4TNB	GRK5	P34947	29.00	-37.00	28.00
4TVJ	PARP2	Q9UGN5	23.00	-1.00	18.00
4UXQ	FGFR4	P22455	-1.00	0.00	15.00
4W9W	BMP2K	Q9NSY1	5.00	-20.00	-46.00
4WSQ	AAK1	Q2M2I8	9.00	-13.00	-50.00
4XCS	PRDX1	Q06830	-32.00	-25.00	-24.00
4YHJ	GRK4	P32298	33.00	29.00	37.00
4YLZ	LGALS4	P56470	-4.00	-24.00	5.00
4Z07	PRKG1	Q13976	29.00	-24.00	-23.00
4ZSG	MAPK7	Q13164	-4.00	-33.00	-7.00
5BVW	DDR1	Q08345	14.00	39.00	41.00

5C8W	PRKG2	Q13237	-5.00	-16.00	-5.00
5D9K	RPS6KA3	P51812	-10.59	17.09	29.19
5DG1	LGALS2	P05162	21.00	157.00	562.00
5DN3	AURKA	O14965	28.00	80.00	8.00
5E1S	INSR	P06213	0.00	20.00	22.00
5FBO	HDAC8	Q9BY41	-26.00	2.00	-14.00
5FCW	PDGFRA	P16234	37.00	16.00	122.00
5GRN	HDAC1	Q13547	-9.00	3.00	-12.00
5ICN	PRDX2	P32119	-49.21	35.36	-4.13
5IWG	HDAC2	Q92769	65.00	29.00	-5.00
5J06	CD33	P20138	-5.00	36.00	14.00
5J3S	TDP2	O95551	7.00	-3.00	14.00
5LCZ	GSTA1	P08263	29.59	6.09	17.19
5LHD	ANPEP	P15144	24.00	10.00	52.00
5M6U	PIK3CD	O00329	36.00	17.00	35.00
5O7P	ERBB3	P21860	69.00	82.00	31.00
5QBU	CTSS	P25774	43.59	-8.91	46.19
5T89	VEGFR1	P17948	-54.00	51.00	60.19
5UJB	ALB	P02768	27.00	10.00	14.00
5UKJ	PIK3CA	P42336	1.00	-11.00	-17.00
5VL3	CD22	P20273	4.00	-4.00	4.00
5WT9	PD1	Q15116	49.00	-48.00	-20.00
5XG8	LGALS13	Q9UHV8	-7.00	-13.00	20.11
5XRG	LGALS10	Q05315	24.00	-6.00	13.00
5XY1	LYN	P07948	-17.00	13.00	-17.00
5YQX	BRD4	O60885	-10.00	-2.00	9.00
5YR4	METAP1	P53582	17.00	8.00	19.00
6AL5	CSNK2A1	P68400	27.00	9.00	71.00
6AX4	CD19	P15391	-15.00	24.00	65.00
6BFN	PLK1	P53350	-23.00	14.00	7.00
6CIC	IRAK1	P51617	119.00	249.00	353.00
6CZK	NNOS	P29475	26.00	33.00	25.00
6EZP	CTSH	P09668	1.00	8.00	-16.00
6FYV	CLK4	Q9HAZ1	-30.00	23.00	-16.00
6FYZ	HDAC4	P56524	-8.00	0.00	13.00
6G0W	PARP14	Q460N5	16.00	-9.00	-23.00
6GU2	CDK1	P06493	328.00	212.00	192.00
6I6Z	CPA1	P15085	-12.00	14.00	-18.00
830C	MMP13	P45452	-2.00	29.00	56.00
